# Exploring the Potential of Oleanolic Acid Dimers–Cytostatic and Antioxidant Activities, Molecular Docking, and ADMETox Profile

**DOI:** 10.3390/molecules29153623

**Published:** 2024-07-31

**Authors:** Andrzej Günther, Przemysław Zalewski, Szymon Sip, Barbara Bednarczyk-Cwynar

**Affiliations:** 1 Department of Organic Chemistry, Faculty of Pharmacy, Poznan University of Medical Sciences, Collegium Pharmaceuticum 2 (CP.2), Rokietnicka Str. 3, 60-806 Poznan, Poland; bcwynar@ump.edu.pl; 2 Department of Pharmacognosy and Biomaterials, Faculty of Pharmacy, Poznan University of Medical Sciences, Collegium Pharmaceuticum 1 (CP.1), Rokietnicka Str. 3, 60-806 Poznan, Poland; pzalewski@ump.edu.pl (P.Z.); szymonsip@ump.edu.pl (S.S.); 3 Department of Pharmacology and Phytochemistry, Institute of Natural Fibres and Medicinal Plants, Wojska Polskiego 71b, 60-630 Poznan, Poland; 4 Center of Innovative Pharmaceutical Technology (CITF), Rokietnicka Str. 3, 60-806 Poznan, Poland

**Keywords:** triterpenes, oleanolic acid, triterpene dimers, oleanolic acid dimers, cytostatic activity, antioxidant activity, ADMETox

## Abstract

The presented work aimed to explore the potential of oleanolic acid dimers (OADs): their cytostatic and antioxidant activities, molecular docking, pharmacokinetics, and ADMETox profile. The cytostatic properties of oleanolic acid (**1**) and its 14 synthesised dimers (**2a**–**2n**) were evaluated against 10 tumour types and expressed as IC_50_ values. Molecular docking was performed with the CB-Dock2 server. Antioxidant properties were evaluated with the CUPRAC method. ADMETox properties were evaluated with the ADMETlab Manual (2.0) database. The results indicate that the obtained OADs can be effective cytostatic agents, for which the IC_50_ not exceeded 10.00 for many tested cancer cell lines. All OADs were much more active against all cell lines than the mother compound (**1**). All dimers can inhibit the interaction between the 1MP8 protein and cellular proteins with the best results for compounds **2f** and **2g** with unsaturated bonds within the linker. An additional advantage of the tested OADs was a high level of antioxidant activity, with Trolox equivalent for OADs **2c**, **2d**, **2g**–**2j**, **2l**, and **2m** of approximately 0.04 mg/mL, and beneficial pharmacokinetics and ADMETox properties. The differences in the DPPH and CUPRAC assay results obtained for OADs may indicate that these compounds may be effective antioxidants against different radicals.

## 1. Introduction

Compounds of natural origin have been the subject of interest for scientists worldwide for years [1]. This interest is related to three aspects: (i) because of known or potential directions of pharmacological activity, (ii) because of potential utilitarian properties, and (iii) because of the possibility of carrying out numerous and various types of chemical transformations leading to new derivatives not yet described in the scientific literature. These derivatives may show similar or entirely new directions of pharmacological activity as their parent compounds.

Plant raw materials and their chemicals have been used as remedies for many diseases since the beginning of human history. The first medical use of medicinal plants probably occurred in Mesopotamia and dates back to 2600 BC [2]. Over the following centuries, knowledge about the medical use of natural products increased, and natural product preparations became increasingly popular. In recent years, there has been a rapid return to natural medicine and medical preparations based on substances of natural origin, both isolated from natural raw materials and chemically modified.

Natural products are used to treat various diseases and are becoming essential for drug discovery and research. However, using substances of non-natural origin is associated with specific challenges, such as the methods of isolating the substances from material, the identification of bioactive substances, the effectiveness of their action, toxicity, the mechanism of their action, and bioavailability [3]. Despite these disadvantages, compounds of natural origin have provided new and potential leads to cancer chemotherapy, and many of them are the drug of choice in cancer treatment [4]. Natural products are important sources of chemical structures, which will be used as templates for constructing new compounds with improved biological properties [5].

**Figure 1:**
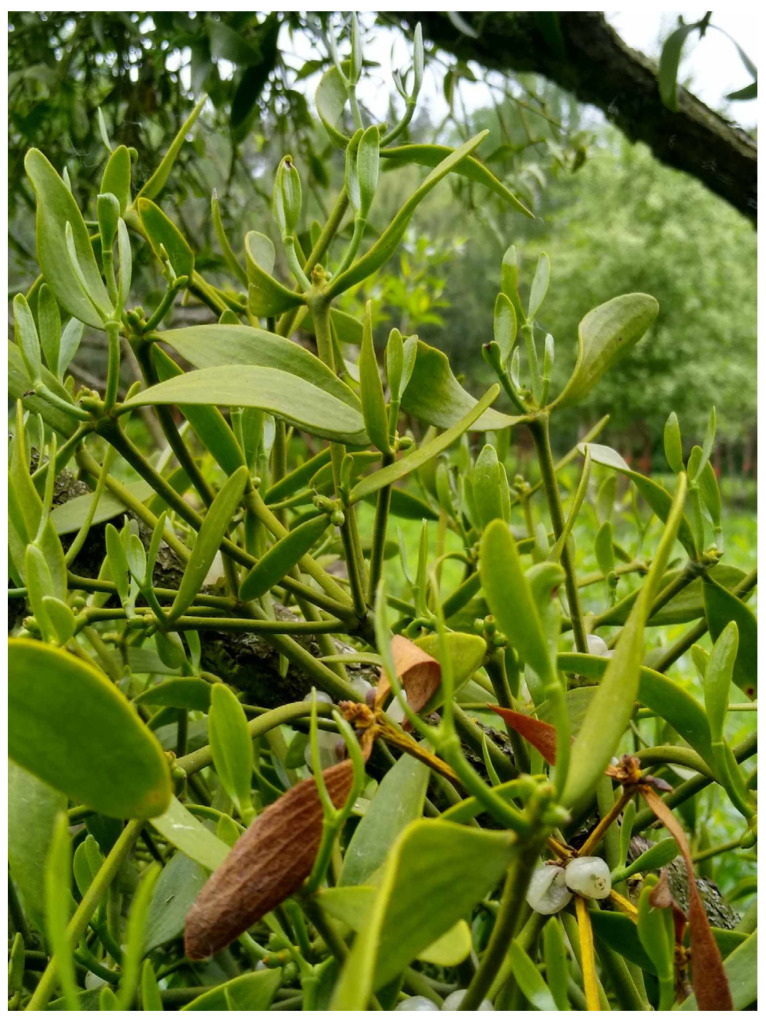

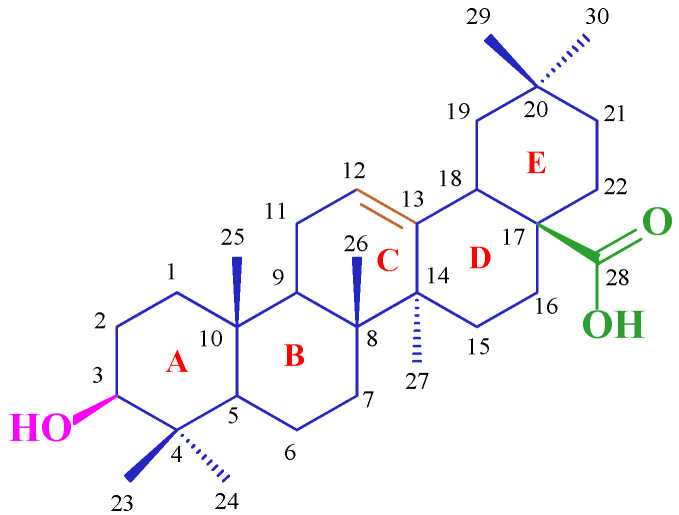
One of the sources of oleanolic acid (**1**)—the mistletoe (*Viscum alba*) herb and the structure of this compound.

Among chemical compounds of natural origin, alkaloids, flavonoids, and terpenoids [6] are particularly interesting. The last of the mentioned groups, also known as isoprenoids, is a vast group of compounds widely distributed in the plant world, occurring as numerous glycosides and in free form. Several subgroups can be distinguished within the over 40,000 terpenoids [7], such as monoterpenoids, diterpenoids, sesquiterpenoids, triterpenoids, and tetraterpenoids. The largest and most famous subgroups mentioned are triterpenoids—compounds whose carbon skeleton comprises 30 carbon atoms. Because of the similarity of the carbon skeleton structure, triterpenoids are divided into several smaller groups, such as oleananes, ursanes, lupanes, friedelanes, and others. The most representative of the first group mentioned, oleananes, which is the most numerous and most widespread among triterpenoids, is oleanolic acid (Figure 1). The presence of this compound has been demonstrated in at least 1600 species of edible and medicinal plants [8]. A rich source of this compound is, among others, the mistletoe herb (*Viscum alba*, Figure 1). The carbon skeleton of oleanolic acid (**1**), as presented in Figure 1, comprises five six-carbon rings, explicitly connected. Figure 1 shows the numbering of individual rings and all carbon atoms in the molecule of the mentioned chemical compound.

Triterpenoids, including oleanolic acid, are becoming more and more popular among scientists around the world. This interest resulted in numerous scientific publications on synthesising new oleanolic acid derivatives and on the pharmacological activity. So far, it has been proven that the mentioned triterpene has, for example, antioxidant [9], antileishmanial [10], antibacterial and antiparasitic [11], antidiabetic [12], antiviral [13], antihypertensive, antiatherosclerotic and antioxidant [14], neuroprotective [15], hepatoprotective [16], anticancer [17], and other activities.

The greatest hopes for oleanolic acid (**1**) are associated with the activity referred to as “anticancer” (antitumour, cytotoxic against cancer cells, cytostatic against cancer cells, etc.). Cancer is the second cause of death worldwide, after cardiovascular diseases, and the trend in cancer incidence and mortality is increasing [18]. So far, a high level of anticancer activity of oleanolic acid (1) has been demonstrated against, e.g., human breast cancer MCF-7 [19], melanoma [20], ovarian carcinoma [21], lung cancer cell lines [22], hepatocellular carcinoma cell line HuH7 [23], and many others. Numerous attempts are also being made to synthesise new derivatives of oleanolic acid (**1**) in order to obtain effective, non-toxic anticancer agents. Also, in our Department, the leading research direction is the synthesis of new oleanolic acid derivatives (**1**) with expected high activity against various cancer cell lines. Experimental work published so far demonstrates a high level of cytostatic activity, primarily against KB, MCF-7, HeLa, Hep-G2, and A-549 cancer cell lines [24,25,26,27,28,29].

One of the factors that plays an essential role in the pathogenesis of cancer (but also many other diseases) is oxidative stress. In many such cases, treatment with oleanolic acid (**1**) has been discovered to be beneficial [9]. The reason for oxidative stress is excessive production of Reactive Oxygen Species (ROS, also known as free radicals) or their insufficient use by the body during numerous life processes, such as respiration and some cell-mediated immune functions [30]. Excess ROS can react with many biomolecules such as DNA [31], lipids [32], and proteins [33], initiating the peroxidation of membrane lipids, leading to the accumulation of lipid peroxides and the damage of DNA and proteins, and finally resulting in disease conditions. The antioxidant effect of oleanolic acid (**1**) probably involves quenching ROS, inhibiting lipid peroxidation, or indirectly stimulating cellular antioxidant defences [33]. Several triterpenoid compounds and their derivatives have been shown to demonstrate promising antioxidant properties in experimental and clinical studies, mainly from the ursane, oleanane, and lupane groups (e.g., [34,35,36,37]).

Our previous publication showed that the connection of two oleanolic acid residues through the C-17 carboxyl group with unbranched dihalogenoalkanes led to compounds with a high cytotoxic and antioxidant activity level. This was indicated by a low IC_50_ value, often below 10 micromoles, and significantly higher Trolox equivalents than the mother compound. Dimers with short chains (up to four carbon atoms) were particularly active. Encouraged by these results, we decided to confirm the antioxidant activity of these compounds using another test. We also decided to assess the level of cytostatic activity using computer methods, perform molecular tests, and evaluate ADMETox parameters using computational methods. The presented work is the first to present biological and computational studies for such a wide range of OADs.

While previous studies have examined methods of OAD synthesis as well as their physicochemical properties, SAR analysis, cytotoxic activity, SI, and antioxidant activity as developed with the DPPH assay, there remains a significant gap in understanding, e.g., how the length of the bridge connecting two triterpene units in OADs influences their cytostatic activity against a larger number of cell lines, their antioxidant activity measured with the CUPRAC method, and their interaction with specific proteins. This study aims to address this gap by calculating IC_50_ values for 74 cancer cell lines and evaluating their antioxidant activity using another assay, as well as their interaction with FAK kinase through molecular docking studies. By elucidating the relationship between bridge length, antioxidant activity, and protein interaction, this study will provide valuable insights into the development of more potent and targeted oleanolic acid-based cytostatics and antioxidants for therapeutic applications.

The objectives of the presented paper were the following: (i) to determine the relationship between the bridge length of OADs and their cytotoxic activity, (ii) to evaluate the antioxidant activity of OADs with different bridge lengths, (iii) to investigate the interaction of OADs with target proteins, like FAK kinase, through molecular docking studies, and (iv) to determine the relationship between the bridge length of OADs and their predicted ADMETox parameters. By achieving these objectives, this study aims to provide insights into designing more effective OADs for therapeutic applications, particularly in areas where antioxidant and/or cancer protein inhibition properties are desired.

## 2. Results

### 2.1. Synthesis of OADs

The structures of oleanolic acid dimers (OADs) **2a**–**2n** tested in our work are provided in Figure 1.

### 2.2. Potential Cytostatic Properties of OADs

**Figure 2:**
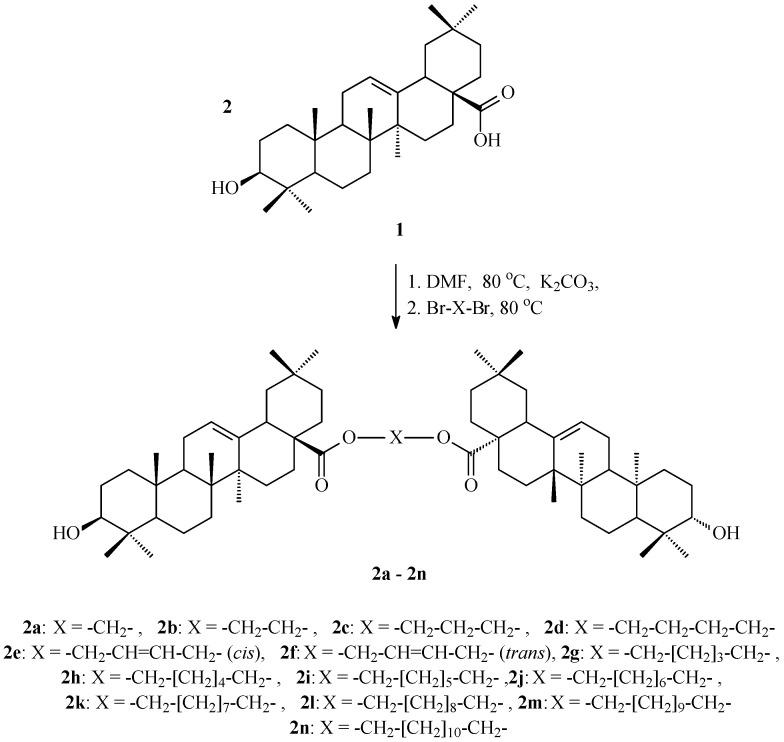
Synthesis of OADs **2a**–**2n**.

The potential cytostatic properties of oleanolic acid (mother compound, **1**, Figure 1) and 14 synthesised oleanolic acid dimers (OADs, **2a**–**2n**, Figure 2) were evaluated against 10 tumour types: breast cancers, central nerve system cancers, colon cancers, leukaemia, melanoma, non-small-cell lung cancers, ovarian cancers, prostate cancers, renal cancers, and small-cell lung cancers. The half-maximal inhibitory concentration (IC_50_) for the triterpenes **1** and **2a**–**2n** are presented in Table 1.

### 2.3. Molecular Docking

#### 2.3.1. Detecting Cavities

**Figure 3:**
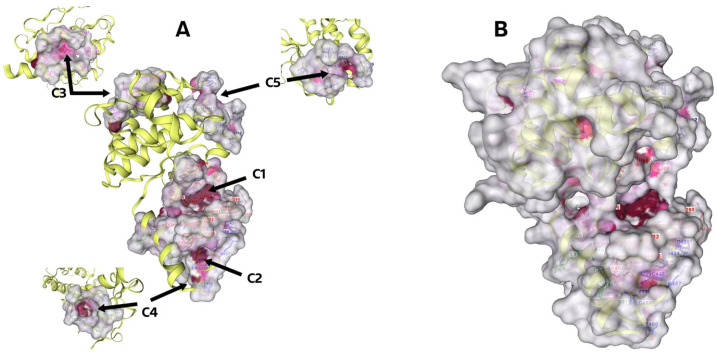
(**A**) Graphical result of searching the top 5 cavities for FAK (PBD ID: 1MP8), where the largest is C1 and the smallest is C5. In addition, a different angle is shown to be better for the C3, C4, and C5 cavities. (**B**) FAK molecule, the dark pink colour indicates the cavity of the molecule.

The CB-Dock2 web server searches concave surfaces for cavities (method is called CurPocket) [39]. Below are the results for the crystal structure of Focal Adhesion Kinase, FAK (PDB ID: 1MP8), whose cavities are highlighted in Figure 3. The top 5 cavities were chosen as candidates for blind docking, ranked based on their size from the largest, C1, to the smallest, C5, as presented in Table 2. Cavities C1–C5 of FAK are presented in the Supplementary Materials (SM.01, Figures S1–S5).

#### 2.3.2. Molecular Docking

According to Table S1 in the Supplementary Material (SM.01), we can see that docking occurs in all cavities (C1–C5). The optimal outcomes have been collated in Table 3. The best results in cavity C1, which is the largest and is calculated to have a volume of 818 (Å^3^), were obtained for dimer **2f**, whose Vina score was −11.6 kcal⋅mol^−1^. Subsequently, superior outcomes were obtained for the C2 cavity with dimer **2e** presenting a Vina score of −8.6 kcal⋅mol^−1^.

**Figure 4:**
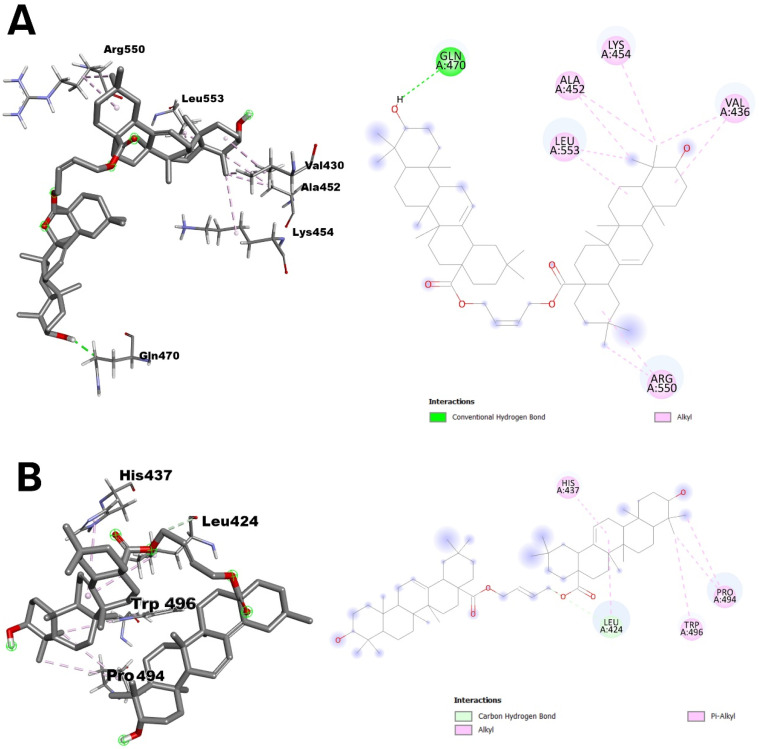
Interactions between structure of 1MP8 and dimers **2f** (**A**) and **2e** (**B**); views: left in 3D, and right 2D. **Legend**: Interactions of dimers **2f** (**A**) and **2e** (**B**) with respective C1-C5 cavities.

Dimer **2f** shows significant hydrogen bonding with the amino acid GLN A:470, as well as being engaged in an alkyl interaction with the amino acids VAL A:436, ALA A:452, LYS A:454, LEU A:553, and ARG A:550, as shown in Figure 4A.

In the C2 cavity, the second largest in the molecule, an effective docking of the dimer **2f** was observed, which, due to an unsaturated bond at the C12–C13 carbon atoms, forms π-alkyl interactions with histidine at position 437 and leucine at position 424. In addition, it establishes a carbon–hydrogen bond with the amino acid LEU A:424 and alkyl interactions with PRO A:494 and TRP A:496, as shown in Figure 4B.

### 2.4. Antioxidant Activity of OADs

**Figure 5:**
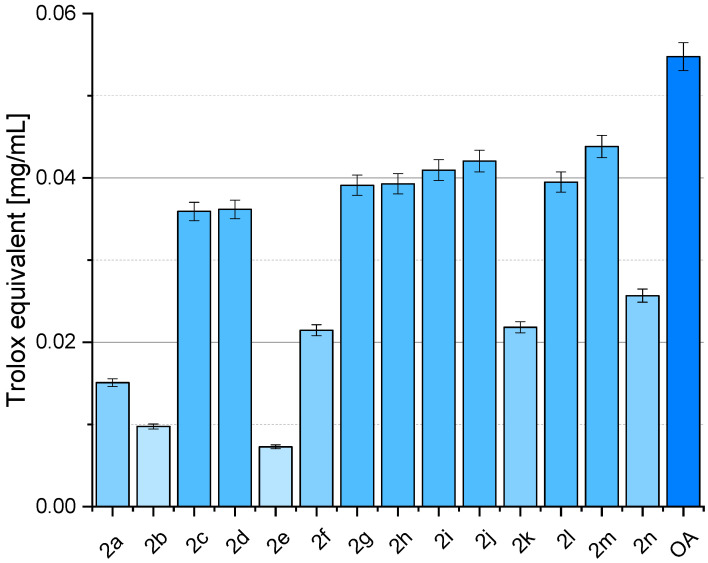
Antioxidant activity in CUPRAC assay of OADs **2a**–**2n** and oleanolic acid (**1**, OA) expressed as Trolox equivalent.

**Figure 6:**
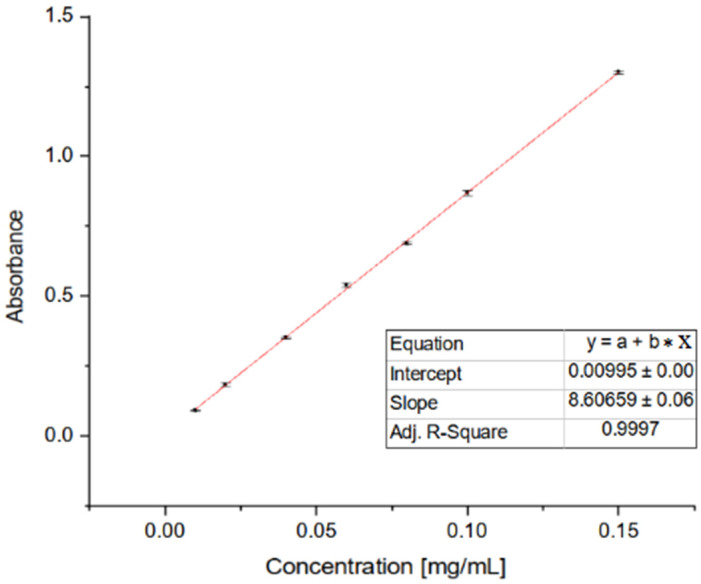
Standard curve for CUPRAC assay as Trolox equivalent.

Figure 5 depicts various samples’ CUPRAC radical scavenging activity, with OA (oleanolic acid, **1**) representing the naturally occurring compound and subsequent entries representing synthesised OADs **2a**–**2n**. The results were presented as % inhibition of the copper(II) ions as Trolox equivalent values, calculated from the standard curve (Figure 6). Activity assays were performed in 8 repetitions.

### 2.5. ADMETox Analysis

The detailed results of the *ADMETox* analysis are provided in the Supplementary Materials (SM.02, Table S1).

**Figure 7:**
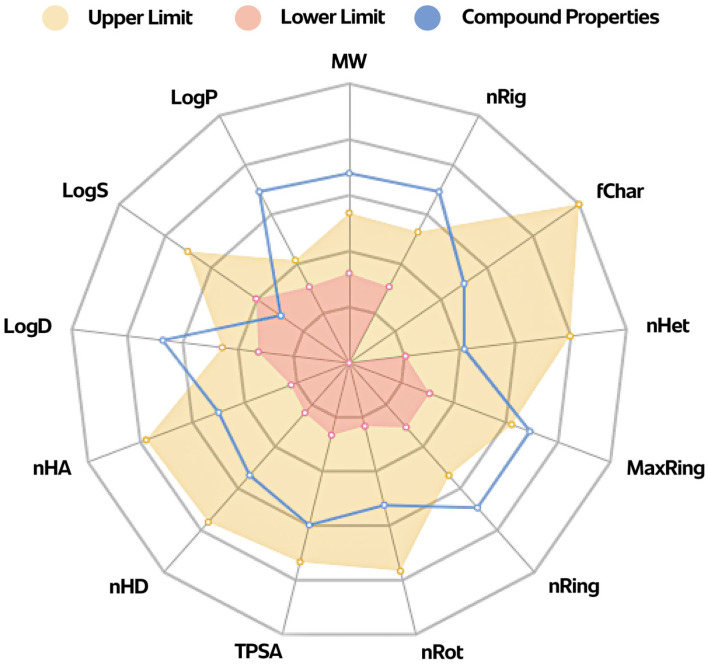
The Physicochemical Properties Diagram for an example dimer **2a**, with the shortest bridge.

The Physicochemical Properties Diagram for an example dimer **2a**, with the shortest bridge, is presented in Figure 7.

## 3. Discussion

### 3.1. Synthesis of OADs

All oleanolic acid dimers (OADs) were obtained from oleanolic acid (**1**) with the application of the method we developed [29]. In short, the synthesis of OADs **2a**–**2n** (Figure 2) included dissolving 1.0 mmol of oleanolic acid (**1**) in DMF, mixing and heating it at approximately 80 °C, and adding a two-fold excess of K_2_CO_3_. After half an hour of further stirring and heating, 0.5 mmol of α,ω-dibromoalkane/α,ω-dibromoalkene was added and the content of the flask was further stirred and heated for another half an hour. The cooled mixture after reaction was poured into approximately five-times the volume of water and slightly acidified with diluted HCl. The resulting white precipitate was filtered off, washed with water to neutralize the pH of the filtrate, dried and crystallised from ethanol or ethanol with water, or re-precipitated with water from an ethanolic solution.

Our previous work has discussed the details regarding the preparation and purification of OADs and their spectral characteristics, relative polarity, and susceptibility to crystallisation [29].

### 3.2. Potential Cytostatic Properties of OADs

The compounds are considered active against cancer cells if the IC_50_ and IG_50_ values are equal or less than 10,000 nM (≤10 µM). Chemical compounds are moderate cytotoxic or cytostatic agents if they present IC_50_ or IG_50_ values in the range of 10 µM < IC_50_ ≤ 30 µM [40,41].

Of the 15 triterpenes (oleanolic acid, mother compound, **1**) and 14 oleanolic acid dimers (OADs, **2a**–**2n**) tested, all or almost all compounds were active against 74 cancer cell lines (Table 1).

Taking into account the IC_50_ value predicted with the application of the pdCSM-cancer program and the structure of the tested ODs, several regularities can be distinguished:

- All OADs **2a**–**2n** will probably show a much higher level of cytostatic activity in in vivo tests than the parent oleanolic acid (**1**), for which only a few lines had an IC_50_ value ≤ 10 µM (lines: MDA-MB-468, XF-498, K-562, P388-ADR, P388, HOP-92, RXF-393, and SN12K1);
- Only in the case of four cancer cell lines did the combination of two oleanolic acid residues into a dimer derivative cause a decrease in the IC_50_ value, i.e., a decrease in cytotoxic activity: SNB-78, M14, M19-MEL, MDA-N, SKA-MEL-5, A-549-ATCC, and 786-0; for all remaining 69 tumour cell lines, the pdCSM-cancer program predicted the increase in cytostatic activity level;
- In the case of more than ten cancer cell lines (e.g., T-47D, SF-295, COLO-205, HT29, RPMI-8226, MALME-3M, NCI-ADR-RES, OVCAR-3, and others), it is beneficial to combine two oleanolic acid residues with a linker longer than the one-carbon one;
- It is not possible to clearly state what effect the presence of a double bond, *cis* or *trans*, in the four-carbon bridge has on the IC_50_ value—the number of cancer cell lines for which the IC_50_ value significantly increases or decreases is similar;
- The tested dimer derivatives of oleanolic acid (OADs **2a**–**2n**) may probably be highly effective cytostatic agents against BT-549, HS-578T, MCF-7, HCC-2998, SW-620, K-562, RMPI-8226, SK-MEL28, HOP-18, HOP-92, NCI-ADR-RES, DU-145, RXF-393, RXF-631, DMS-114 (IC_50_ in the range of 1.01–5.00 µM), MDA-MB -468 (IC_50_ in the range of 0.10–0.99 µM), and P388-ADR, P388, SN12K1, and SN12K1 (IC_50_ ≤ 0.09 µM);
- It is hard to find a clear relationship between the structure of OADs and the level of their cytostatic activity. Probably the reason for the lack of this dependence may be the geometry of the OAD molecule—the length of the linker and the presence (or lack) of a double bond, and, even more importantly, the type of this unsaturated bond (*cis*/*trans*) may cause a given dimer to better or worse adapt to the enzymes of cancerous cells. The structure of the linker most likely involves a specific mutual arrangement of two triterpene residues, and these arrangements may be different—e.g., two triterpene residues may be arranged in a straight line, or one below the other, or in still other ways. Therefore, some dimers fit better into the enzyme pocket, while others fit worse.

### 3.3. Molecular Docking

CB-Dock2 is a highly efficient and accurate molecular docking program that has gained widespread recognition in the scientific community for its ability to accurately predict the binding poses and affinities between small molecules and target proteins. Compared to other docking programs, CB-Dock2 offers several key advantages that make it a superior choice for drug discovery and development applications.

One of the primary advantages of CB-Dock2 is its ability to accurately account for the flexibility of both the ligand and the target protein during the docking process [42]. This is a critical feature, as the ability of a small molecule to adopt different conformations and the potential for induced-fit binding interactions between the ligand and the target protein can have a significant impact on the binding affinity and the overall efficacy of a drug candidate. In contrast, many other docking programs rely on rigid body docking, which can fail to capture these important dynamic interactions and lead to inaccurate predictions of binding poses and affinities.

Another key advantage of CB-Dock2 is its use of an ensemble-based docking approach, which incorporates multiple conformations of the target protein generated through molecular dynamics simulations [43]. This approach allows for the capture of the inherent flexibility and dynamics of the target protein, which can have a significant impact on the binding of small molecules. By considering a range of possible protein conformations, CB-Dock2 is able to provide more accurate predictions of binding poses and affinities and is less susceptible to the limitations of rigid-body docking approaches.

Moreover, CB-Dock2 employs advanced scoring functions and optimisation algorithms that enable it to accurately predict the binding affinity of small molecules to target proteins [42]. This is particularly important in the context of drug discovery, where the ability to accurately predict the binding affinity of candidate compounds can greatly facilitate the identification and optimisation of lead compounds.

The superior performance of CB-Dock2 has been extensively validated through a variety of studies, which have consistently demonstrated its ability to outperform other leading docking programs in terms of both accuracy and computational efficiency [44,45]. These studies have shown that CB-Dock2 is able to generate more accurate binding poses and more reliable predictions of binding affinities, making it a highly valuable tool for a wide range of drug discovery and development applications.

The FAK protein (PDB ID: 1MP8) was chosen for this study due to its well-characterised structure and its central role in various signalling cascades that drive cancer development and progression [46,47,48]. Previous research has identified a known cavity in the FAK structure that serves as a target for inhibitors [49]. This cavity, located in the kinase domain, has been the focus of numerous efforts to develop small-molecule inhibitors that can disrupt FAK-mediated signalling and potentially suppress tumour growth and metastasis.

The crystal structure of FAK (PDB ID: 1MP8) has been extensively studied and provides valuable insights into the structural features and potential druggable sites within the protein. Structural analysis of FAK can inform the design of novel inhibitors that can selectively target this kinase and modulate its activity in cancer cells. Furthermore, understanding the endogenous control mechanisms and interaction partners of FAK, as discussed in the literature [49], can shed light on alternative strategies for regulating its function in the context of cancer development. The recent developments in PFKFB3 inhibitors, a protein closely related to FAK, have also provided valuable insights into the potential of targeting key metabolic enzymes involved in tumour progression [50]. These findings suggest that a combined approach targeting both FAK and associated metabolic pathways may offer a more comprehensive strategy for cancer therapy.

FAK (Focal Adhesion Kinase) is a cytoplasmic tyrosine kinase located in cells that form junctions with the extracellular matrix (ECM) or other cells. Its primary function is to transduce signals from integrin receptors to the intracellular protein cascade, indirectly affecting many cellular processes such as cell cycle regulation, adhesion, migration, invasion, metastasis, cytoskeletal protein phosphorylation, and apoptosis. Deregulation of FAK function is a critical component of tumour progression. FAK expression varies with tumour stage, increases during the invasive phase, and correlates with cancer cell migration, invasion, and metastasis. The level of FAK expression can be used as a prognostic indicator of tumour malignancy [51].

A study of the crystal structure of FAK (PDB ID: 1MP8) was carried out to identify potential pockets on the protein’s surface using a CB-Dock2 server, allowing molecular docking to assess the interaction of different dimers with these pockets. These results are relevant to the search for new inhibitors of the FAK protein with potential therapeutic applications.

Using the CurPocket method, the CB-Dock2 server identified the five most significant pockets in the 1MP8 structure. These pockets were ranked by volume from largest (C1) to smallest (C5). The most oversized pocket (C1) had a volume of 818 Å³, while the most petite pocket (C5) had a volume of 84 Å³. The locations and sizes of these pockets are detailed in Table 2 and Figure 3.

Molecular docking showed that all the pockets analysed (C1–C5) could accept different dimers with different Vina scores, which indicate the strength of ligand binding to the protein. The best docking results were obtained for dimer **2f** in pocket C1, confirmed by a Vina score of −11.6 kcal/mol. This was followed by dimer **2e** in the C2 pocket with −8.6 kcal/mol.

The lower the affinity (Vina score), the stronger the binding interaction between the molecules, so all dimers showed good docking results and can inhibit the interaction between the 1MP8 protein and cellular proteins. This modelling did not answer whether the length of the carbon chain—the linker—influences the interaction between the protein and dimer. The best result was obtained for dimer **2f**, which is in the *trans* configuration, and the linker has four carbon atoms, including a double bond, and for the C2 pocket, dimer **2g,** which is in the *cis* configuration, with the same linker.

Molecular docking to pockets identified on the surface of FAK kinase revealed diverse and robust interactions of different dimers with the protein, suggesting that some of these compounds may be promising FAK inhibitors. The best results were obtained for the dimer **2f** in the largest pocket (C1), suggesting that larger pockets on the protein surface may provide more stable and potent binding sites for ligands.

### 3.4. Antioxidant Activity of OADs

The CUPRAC assay, known for its ability to assess total antioxidant capacity, yielded a different perspective on the antioxidant potential of these compounds compared to the DPPH assay conducted in a previous publication [29]. Oleanolic acid (OA, **1**, Figure 1) exhibited higher antioxidant activity in the CUPRAC assay than in the DPPH assay, suggesting that OA might possess a broader spectrum of antioxidant mechanisms that are more effectively captured with the CUPRAC method. This discrepancy underscores the importance of employing multiple assays to evaluate antioxidant activity comprehensively. Several synthesised derivatives demonstrated varying degrees of antioxidant activity in both assays. For example, compound **2a** showed high activity in the DPPH assay [29] but much lower activity in the CUPRAC assay, while compounds **2g**, **2i**, **2j**, and **2m** demonstrated substantially higher antioxidant activity in the CUPRAC assay. Compound **2f** exhibited relatively consistent antioxidant activity across both assays, indicating that its mechanism of action may be versatile, effectively contributing to both hydrogen donation and overall reducing capacity. These results highlight significant variance in antioxidant activity between the two assays for certain derivatives, suggesting distinct antioxidant mechanisms that interact variably with the reactive species and conditions present in each assay.

The differing outcomes between the DPPH and CUPRAC assays suggest that the synthesised derivatives of oleanolic acid exhibit distinct antioxidant mechanisms. The DPPH assay primarily measures the ability of antioxidants to donate hydrogen atoms to the DPPH radical, while the CUPRAC assay evaluates the overall reducing capacity, including the ability to reduce copper(II) ions to copper(I). Compounds such as **2g**, **2i**, **2j**, and **2m** (Figure 2) demonstrated robust reducing capacity in the CUPRAC assay, indicating they possess functional groups or structural features that facilitate electron transfer more effectively than hydrogen donation. Conversely, compound **2a**, which showed the highest DPPH activity [29], exhibited significantly lower activity in the CUPRAC assay, suggesting a specific affinity for DPPH radicals rather than a broad-spectrum reducing capability. The complementary use of DPPH and CUPRAC assays provides a more comprehensive understanding of the antioxidant properties of oleanolic acid and its derivatives. While the DPPH assay emphasises hydrogen atom donation, the CUPRAC assay highlights overall reducing power. The variability in results between the two assays underscores the importance of employing multiple methodologies to fully elucidate these compounds’ antioxidant mechanisms and potential therapeutic applications. Notably, compounds such as **2g**, **2i**, **2j**, and **2m** show promising enhanced activity in the CUPRAC assay (Figure 5), warranting further investigation into their structural features and potential applications in oxidative stress-related conditions.

Mechanistic differences between the assays lead to varying antioxidant activity profiles for the same compounds. The DPPH assay primarily captures hydrogen atom transfer (HAT) mechanisms, while the CUPRAC assay encompasses both HAT and single electron transfer (SET). Environmental and experimental conditions, such as differences in solvent polarity, pH, and other assay conditions, also influence the observed activity. Certain functional groups may be more active under specific conditions present in one assay but not the other. The structural features of OADs play a crucial role in their antioxidant mechanisms. For instance, compounds **2g**, **2i**, **2j**, and **2m** (Figure 2) likely have electron-rich functional groups that facilitate SET, explaining their high activity in the CUPRAC assay (Figure 5).

The complementary use of DPPH and CUPRAC assays provides a more thorough understanding of the antioxidant properties of oleanolic acid and its derivatives. Employing multiple assays is crucial for elucidating the full spectrum of antioxidant mechanisms. Compounds showing high activity in the CUPRAC assay, such as **2g**, **2i**, **2j**, and **2m**, warrant further investigation for their potential applications in oxidative stress-related conditions. Their robust SET mechanisms could be particularly beneficial in scenarios requiring robust reducing capacity. Future studies should focus on detailed mechanistic analyses and structure–activity relationships to optimise the antioxidant properties of oleanolic acid derivatives. Additionally, in vivo studies and clinical trials will be essential to validate their therapeutic potential.

The assessment of antioxidant capacity is a crucial aspect in the evaluation of food and natural product quality. Two widely used methods for this purpose are the 2,2-diphenyl-1-picrylhydrazyl and the cupric ion reducing antioxidant capacity assays [52]. While both techniques aim to measure the ability of a compound to scavenge free radicals, discrepancies in their results have been observed, which warrants a more thorough investigation to elucidate the underlying antioxidant mechanisms. The DPPH assay relies on the ability of an antioxidant to donate a hydrogen atom or an electron to the stable DPPH radical, resulting in its reduction and a subsequent colour change. In contrast, the CUPRAC method measures the reducing power of a sample by quantifying its ability to convert the Cu–neocuproine reagent complex into the Cu(I) form [52]. The differences in the nature of the radicals and oxidants involved in these two assays can lead to divergent results, as the affinity of a particular antioxidant may vary depending on the specific reaction mechanism. For instance, the polarity and partition behaviour of the antioxidant compounds within the sample matrix can influence their accessibility and reactivity towards the assay reagents [53]. Additionally, the DPPH assay may be more sensitive to the presence of certain types of antioxidants, such as phenolic compounds, while the CUPRAC method may be more responsive to the reducing capacity of other species, like metal-chelating agents [54]. Furthermore, the kinetics of the antioxidant–radical interactions can also contribute to the discrepancies observed between the DPPH and CUPRAC assays.

In conclusion, the differences in the DPPH and CUPRAC assay results obtained for OADs can be attributed to the distinct oxidation mechanisms and the varying affinities of antioxidants towards the different radical or oxidant species involved. A comprehensive understanding of these underlying factors is crucial for the accurate assessment of the antioxidant capacity and the elucidation of the antioxidant mechanisms in complex natural matrices.

### 3.5. ADMETox Analysis

Chemical modification of chemical substances, both synthetic and of natural origin, is carried out mainly to obtain new derivatives of utilitarian importance. One type of utilitarian properties is the various directions of pharmacological activity. Each newly obtained chemical substance, which is a potential drug candidate, should not only have the desired level of pharmacological activity but also should be safe to use and demonstrate favourable parameters of absorption, distribution in the body, metabolism, and excretion, i.e., it should have a favourable ADMETox profile.

Predictions of ADMETox properties should occur at the early stages of the development of work on a potential drug to increase the chances of high effectiveness and safe use. In order to select among the obtained substances with a proven level of pharmacological activity, those that have the best pharmacokinetic properties and high in vivo bioavailability, the compounds are subjected to screening tests according to the principle of “drug-like soft”. The above rule contains the restriction to molecular weight, logP, hydrogen bond acceptors (HBAs), hydrogen bond donors (HBDs), topological polar surface area (tPSA), etc.

The tested OADs **2a**–**2n** (Figure 2) presented favourable values for most parameters determining the physicochemical properties (e.g., nHA, nHD, nRot, nRing, nHet, fChar, nRig, flexibility, and tPSA (Figure 7; Supplementary Materials, Table S2). Due to the very low solubility of the above compounds in water, the values of logS, logP, and logD were outside the optimal range, or, in a few cases, they were moderately favourable. The QED (quantitative estimation of drug-likeness) test showed that all the tested compounds (**2a**–**2n**) are too complex in terms of structure to be similar to known drugs (QED ≤ 0.203). At the same time, all OADs, **2a**–**2n**, are easy to synthesise as the synthetic accessibility value is about 6. The number of sp^3^ hybridised carbons in the above 14 triterpenes, **2a**–**2n**, is ~0.900 which is a favourable value. The MCE-18 value for all OADs (**2a**–**2n**) exceeds 45, which means a high level of novelty, which follows the trends currently observed in medicinal chemistry. The NP value (natural product-likeness) of about 1.5 confirms the high similarity to compounds of natural origin (from which compounds **2a**–**2n** were obtained). PAINS, BMS, and Chelator tests are negative for almost all tested triterpenes, which means that there are no unfavourable elements of the structure of the molecules of these substances, which can be potentially responsible for toxicity or may, for example, enter into chemical interaction with other chemical substances present in the body. In both Caco-2 and MDCK tests, all triterpenes (**2a**–**2n**) showed good permeability. Theoretical predictions indicate that almost all tested OADs (**2a**–**2n**) will probably bind well to plasma proteins and smoothly penetrate the blood–brain barrier, showing excellent volume distribution (VD, about 1 L/kg) and an acceptable percentage of the fraction unbound to plasma proteins (about 90%). The excretion of the tested triterpenes is predicted by applying CL and T1/2 tests. The clearance of the tested triterpenes (**2a**–**2n**) was in a range of 7–11 mL/min/kg, with zero probability of being short-half-life compounds. Almost all the tested triterpenes tested showed a very low probability of toxicity (in general, below 0.300) and low parameters of biotoxicity.

Taking into account the relationship between the structure of the obtained OADs (**2a**–**2n**) (more precisely: between the length of the bridge connecting two triterpene units) and the predicted level of ADMETox parameters, it is difficult to observe a clear impact of the bridge structure on these parameters. The elongation of the bridge certainly adversely affects the physicochemical parameters, such as molecular weight, nRot, logS, logP, or logD, due to the increase in the polarity of the obtained compound, which is associated with the deterioration of the solubility of the potential drug in water and aqueous solutions. Some medicinal chemistry, distribution, and metabolism parameters are also slightly worse, while many absorption and metabolism parameters, and almost all Toxicity parameters, are improved. Notably, for dimers with the longest chains in the bridge, i.e., containing 10 and more CH_2_ groups, almost all parameters from the Tox21 panel improved significantly, while some absorption, distribution, and metabolism parameters deteriorated. It can be assumed that these parameters have become weaker because of the higher molecular weight of individual OADs and the decrease in their polarity. Comparing the ADMETox parameters for three dimers with a four-carbon bridge, saturated (**2d**), *cis*-unsaturated (**2e**), and *trans*-unsaturated (**2f**), in the case of nearly 20 tested parameters, apparent differences were observed in the probability of occurrence of a given parameter, generally in favour of OADs whose two triterpene units are connected by a bridge containing an unsaturated bond (OADs **2e** and **2f**), preferably in the *cis* arrangement (**2e**).

## 4. Materials and Methods

### 4.1. OADs Preparation

The methods for preparing OADs and their spectral characterisation are provided in our previous paper [29].

### 4.2. Potential Cytostatic Properties of OADs

Cytostatic activity testing was performed using the pdCSM-cancer [38] computer program, using 74 cancer cell lines belonging to 10 types.

### 4.3. Molecular Docking

#### 4.3.1. Ligands Preparation

The preparation of ligands involved initially sketching the two-dimensional (2D) structures of OADs **2a**–**2n** using ChemDraw 22.0.0. Subsequently, these structures were converted into three-dimensional (3D) representations in OpenBabel [39] format to determine the coordinates representing the most energetically favourable conformation. Avogadro version 1.2.0 software facilitated geometry optimisation utilising the force field: Universal Force Field (UFF) with the Steepest Descent algorithm.

The optimised 3D structures of OADs **2a**–**2n** were generated as SDF files and used as input files for docking analysis carried out with CB-Dock2 server.

#### 4.3.2. Protein Preparation

The crystallographic data for the Focal Adhesion Kinase (FAK) protein 1MP8 structure was obtained from the RCSB Protein Data Bank (PDB ID: 1MP8) with a resolution of 1.60 Å. The X-ray crystal structure of FAC is complexed with Adenosine-5′-diphosphate.

The downloaded 1MP8.pdb protein molecule did not undergo preparation—like removing ligands or crystal water molecules and adding missing hydrogen. The CB-Dock2 server performs these steps by itself.

#### 4.3.3. Detecting Cavities and Uploading Ligands

After importing the 1MP8 molecule into the CB2-Dock server, the number of cavities for docking was set to 5 in the ‘more parameters’ option under the Number of cavities for docking field, the email address field was filled in order to receive files, and the Search Cavities function was pressed. The ligands were uploaded (dimers **2a**–**2n**) as well as the 1MP8 protein, the Number of cavities for docking field was set to 5 cavities in the more parameters option, the e-mail address field was filled out, and the Auto Blind Docking button was pressed.

### 4.4. Antioxidant Activity of OADs

The OADs’ antioxidant properties were determined by using the CUPRAC assay, according to Garbiec et al., with modifications [55]. Previously prepared solutions of neocuproine, copper chloride, and ammonium acetate buffer in the same volumes (CUPRAC reagent solution) were added to the volumetric flask wrapped in aluminium foil, and the contents were mixed thoroughly. Then, 50.0 µL of the test sample and 150.0 µL of CUPRAC reagent solution were added to the wells of the 96-well plate. The control sample was a mixture of the CUPRAC reagent and the extraction solvent. The entire experiment was repeated three times, each time in triplicate, which yielded nine results (*n* = 9). The sample plate was wrapped in aluminium foil, shaken for 5 min at 25 °C, then incubated for 30 min at room temperature. Finally, absorbance measurement was performed at a wavelength of 450 nm.

### 4.5. ADMETox

The physicochemical properties, pharmacokinetics, and ADMETox (adsorption, distribution, metabolisms, excretion, and toxicity) activity of compounds **2a**–**2n** were estimated based on the comprehensive database ADMETlab Manual (2.0) [56]. First, the structures of the analysed compounds were prepared using the JSME editor.

## 5. Conclusions

Connecting two oleanolic acid residues with unbranched bridges of various lengths, including those containing a *cis*- or *trans*-unsaturated bond, results in obtaining dimer derivatives **2a**–**2n** (oleanolic acid dimers, OADs) with a high level of cytostatic activity against the selected cancer cell lines, which was proven in our previous publication [29]. The effectiveness of the obtained OADs as anticancer agents was also demonstrated in this publication, using computer calculations. For the vast majority of the cancer cell lines used, the program predicted a very favourable IC_50_ value, generally exceeding 5000, which means that the obtained OADs, especially those with short bridges (preferably containing four or less carbon atoms in the bridge: **2a**–**2f**) can become drug candidates. Molecular docking performed for the obtained OADs **2a**–**2n** presented the optimal docking outcomes for each ligand cavity with OADs **2e** and **2f**, both with the unsaturated four-carbon linker.

The results of two tests for antioxidant activity for the discussed OADs **2a**–**2n**, i.e., DPPH (results are published in [29]) and CUPRAC (results are presented in this publication) seem to be complementary. The variability in results between the two assays underscores the importance of employing multiple methodologies to fully elucidate these compounds’ antioxidant mechanisms and potential therapeutic applications.

The discussed OADs **2a**–**2n** were characterised by a favourable ADMETox profile, despite a relatively high molecular weight. Although the obtained OADs **2a**–**2n** did not meet the so-called “golden rule of three” (Lipinski Rule, Pfizer Rule, and GSK Rule) criteria, it can be expected that the obtained dimers **2a**–**2n**, especially those with the shortest bridges (up to four carbon atoms, **2a**–**2f**), will be absorbed, distributed, metabolised, and excreted to a sufficiently high extent in vivo and will be non-toxic to healthy tissues and organs.

The relationship between the structure of OADs, especially the length of the linker, and the level of biological activity of these substances is not entirely obvious. However, dimers with shorter bridges are slightly more active, so the subsequent planned work on synthesising new oleanolic acid dimers and testing their activity will mainly concern dimers with short bridges. The second reason in favour of this idea is the fact that OADs are pretty large molecules, and if they had a chance to become drug candidates or even drugs in the future, a significant molecular weight could (but would not have to) be one of the parameters reducing the bioavailability of such a substance.

The conclusions drawn in our study highlight the potential of oleanolic acid dimers as both cytostatic and antioxidant agents. Importantly, these findings have broader implications for drug development and potential clinical applications of the mentioned OADs. The potent cytostatic effects observed, particularly in dimers with shorter bridges, suggest that these compounds could serve as valuable lead candidates for the development of novel anticancer therapies. The demonstrated antioxidant activity of the dimers is also noteworthy, as oxidative stress is a well-established contributor to the pathogenesis of various diseases, including cancer [57].

The modulation of multiple intracellular targets by oleanolic acid and its derivatives, as reported in the literature [58], further underscores the therapeutic potential of these compounds. The ability to fine-tune the bridge length and introduce unsaturated bonds provides an opportunity to optimise the pharmacological properties of the oleanolic acid dimers, potentially leading to the identification of highly potent and selective anticancer agents.

Given the ongoing challenges in cancer therapy, including the development of drug resistance and the unacceptable toxicity profiles of many currently used chemotherapeutics, the discovery of natural-product-based compounds with improved efficacy and safety profiles is of paramount importance [58]. The promising results presented in this paper suggest that oleanolic acid dimers (OADs) warrant further investigation as potential anticancer and antioxidant agents, with the possibility of exploring their utility in combination with existing treatment modalities or as standalone therapies.

Future research concerning OADs will focus on elucidating the precise mechanisms of action underlying the cytostatic and antioxidant activities of the oleanolic acid dimers, as well as evaluating their pharmacokinetic and pharmacodynamic properties in more depth. Additionally, in-depth studies on the bioavailability, metabolism, and potential toxicity of these compounds will be crucial in advancing them toward clinical development.

One major point of concern regarding triterpene compounds like oleanolic acid and its derivatives is their high lipophilicity. This property can lead to various challenges, such as poor water solubility and potential issues with bioavailability and tissue distribution. To address this challenge, researchers have explored potential modifications to reduce lipophilicity without compromising the biological activity of these compounds.

The first strategy involves the modification of the triterpene backbone itself. A study on *Isodon loxothyrsus* identified a new triterpenoid, 3β,13β-dihydroxy-urs-11-en-28-oic acid, which possesses additional hydroxyl groups compared to oleanolic acid [59]. The introduction of these polar substituents can potentially enhance the compound’s solubility and reduce its lipophilicity.

Another approach that has been investigated is the incorporation of polar functional groups into the triterpene structure. For example, the introduction of hydroxyl or carboxyl groups can increase the compound’s polarity and water solubility, potentially improving its pharmacokinetic properties. Researchers have reported the synthesis of 22β-hydroxyolean-12-en-28-oic acid, a derivative of oleanolic acid, which showed promising results in terms of reduced lipophilicity compared to the parent compound [60]. The structure of oleanolic acid and the presence of three reactive functional groups (the C-3 hydroxyl, the C-17 carboxyl, and the C-12–C-13 double bond) allow for numerous chemical modifications, as a result of which a free group can be introduced into the molecule of the parent compound, e.g., hydroxyl or carboxyl group. The simplest example of a reaction allowing for such an action is the addition of the rest of the dicarboxylic acids (succinic, glutaric, phthalic, etc.). Such reactions can also be successfully used for dimeric triterpene derivatives and will be the subject of our future research.

The aim of such chemical transformations will not only be to obtain new derivatives with an interesting structure and more favourable lipophilicity, but we also expect that they will be compounds with high cytotoxic/cytostatic activity against cancer cell lines. Table 4 summarizes, as an example, the IC_50_ results for four oleanolic acid dimers (**2b**, **2d**, **2h**, and **2j**), obtained in our tests and known from the literature. These results show that OADs are compounds with high biological activity, and it is worth making them the subject of further research.

Our previous research on the chemical transformations of triterpenes from the oleanane group shows that that, in order to obtain a derivative with a high level of cytotoxic activity, it is preferable to transform compounds as follows:

- The C-3 hydroxyl into an acetoxy group [24]);
- The C-3 hydroxyl into a keto group and next into oxime [24];
- The C-3 or the C-12 oxime group into its 3,5-dinitrobenzoic derivative [27,28];
- The C-3 hydroxyimino group into the lactam system [25];
- The C-17 carboxyl group into an ester or morpholide group [25].

As can be seen from the cited works, the derivatives obtained in this way are characterised by a high level of cytostatic activity—IC_50_ values for these compounds were often below 5 micromoles. In the near future, we will conduct research on the mechanism of this activity.

## Figures and Tables

**Table 1:** Potential cytostatic properties of OADs **2a**–**2n** and reference compound (**1**) against the tested cell lines determined via pdCSM-cancer [38] and expressed as the half-maximal inhibitory concentration (IC_50_).

≥30.01	20.01–30.00			15.01–20.00			10.01–15.00			5.01–10.00				1.01–5.00			0.10–0.99			0.01–0.09		
							
**Cancer Cell Line**		**IC_50_ (µM)**																				
		**Compound Number**																				
		**1 (OA)**	**2a**		**2b**	**2c**		**2d**	**2e**		**2f**	**2g**	**2h**		**2i**	**2j**		**2k**	**2l**		**2m**	**2n**
**BT-549**		26.42	9.93		3.89	3.89		3.62	6.78		6.78	3.54	3.47		3.43	3.42		3.46	3.47		3.40	3.43
**HS-578T**		16.75	7.43		3.42	3.35		3.29	3.05		3.05	3.46	3.57		3.48	3.52		3.55	3.55		3.49	3.51
**MCF-7**		19.72	5.14		1.73	2.02		1.94	2.87		2.87	2.03	1.72		1.68	1.66		1.52	1.49		1.49	1.40
**MDA-MB-231-ATC**		19.45	7.45		5.44	4.42		5.87	5.44		5.44	5.89	5.912		5.79	5.78		5.78	5.82		5.82	5.85
**MDA-MB-468**		3.40	0.35		0.24	0.63		0.62	0.23		0.23	0.62	0.61		0.61	0.61		0.61	0.61		0.61	0.61
**T-47D**		14.70	14.96		7.80	6.41		8.00	7.03		7.03	7.98	8.15		7.94	7.94		8.07	8.07		8.45	8.45
**SF-268**		21.68	11.67		11.91	10.12		10.18	11.94		11.94	10.28	10.28		10.18	10.16		9.95	9.95		10.05	10.07
**SF-295**		22.86	11.14		9.51	9.40		9.46	10.00		10.00	9.73	5.53		9.55	9.77		10.05	10.05		10.21	9.44
**SF-539**		24.60	12.19		20.04	19.01		18.92	14.19		14.45	18.66	19.41		19.41	20.18		20.46	20.10		20.61	20.51
**SNB-19**		17.82	14.12		10.79	12.79		13.83	17.06		17.06	14.49	14.35		14.12	12.65		11.83	11.45		11.50	11.43
**SNB-75**		27.54	7.38		13.90	13.40		12.59	11.40		11.40	14.86	15.67		15.00	15.45		15.10	15.17		15.35	15.31
**SNB-78**		15.56	15.78		20.41	26.49		26.48	23.93		23.93	26.48	26.67		26.67	26.67		26.67	26.67		26.67	26.67
**U251**		14.55	9.27		9.53	9.64		10.18	9.22		9.22	10.35	9.57		9.77	9.46		9.37	9.22		9.08	8.65
**XF-498**		5.99	3.49		6.08	5.92		5.89	4.82		4.82	5.69	5.57		5.82	6.29		6.34	6.14		6.12	6.18
**COLO-205**		19.01	10.69		9.77	9.79		9.51	8.75		8.75	9.59	9.70		10.00	10.40		10.49	10.49		10.62	10.57
**DLD-1**		14.27	9.62		9.31	9.98		11.91	5.14		5.14	12.39	13.21		13.21	13.46		13.46	13.33		13.03	13.00
**HCC-2998**		15.74	5.32		4.63	4.32		4.47	4.25		4.25	4.25	4.19		4.21	4.27		4.33	4.32		4.23	4.17
**HCT-116**		18.11	1.07		11.56	9.40		10.88	22.23		22.23	11.36	10.57		10.57	10.57		10.62	10.45		10.50	10.45
**HCT-15**		16.90	1.41		19.63	16.98		19.91	17.38		17.38	20.00	20.51		20.51	20.47		19.91	20.00		20.04	20.00
**HT29**		17.26	1.98		13.33	9.59		8.93	9.91		9.91	8.98	9.06		9.12	9.12		9.22	8.95		9.08	9.01
**KM112**		13.09	1.67		11.91	11.30		10.49	25.82		25.82	10.89	10.02		9.12	8.71		9.08	8.55		8.73	8.63
**KM20L2**		11.75	8.14		13.15	13.06		13.06	11.91		11.91	13.77	14.27		14.90	15.03		14.79	15.17		16.94	16.94
**SW-620**		25.06	6.80		4.93	4.76		4.93	5.05		5.05	4.93	4.93		4.93	4.93		4.93	4.90		4.90	4.90
**CCR-CEM**		16.00	9.08		6.59	6.58		6.75	6.25		6.25	6.59	7.40		7.89	8.38		8.47	8.93		9.84	10.07
**HL-60TB**		17.18	10.37		10.94	9.20		10.30	9.20		9.20	10.57	10.47		10.45	10.84		10.84	10.49		10.42	10.47
**K-562**		8.31	3.55		3.92	4.09		4.18	3.93		3.93	4.15	4.31		4.52	4.73		4.64	4.56		4.71	4.73
**MOLT-4**		23.01	21.13		12.97	13.71		13.49	10.00		10.00	14.26	14.96		15.24	15.85		15.99	16.37		16.48	16.44
**P388-ADR**		3.76	0.03		0.08	0.08		0.08	0.08		0.08	0.08	0.08		0.08	0.08		0.08	0.08		0.08	0.08
**P388**		2.39	0.01		0.03	0.02		0.02	0.03		0.03	0.02	0.02		0.02	0.02		0.02	0.02		0.02	0.02
**RPMI-8226**		15.92	7.36		3.53	2.59		2.57	2.55		2.55	2.52	2.41		2.31	2.25		2.15	2.06		1.95	1.89
**SR**		16.25	10.14		13.21	14.29		16.25	12.02		12.02	16.59	18.41		19.23	17.10		15.27	14.93		14.52	12.68
**LOX-IMVI**		18.66	22.23		13.74	13.77		13.74	14.06		14.06	14.35	14.16		14.35	15.03		14.96	15.24		15.49	15.27
**M14**		12.68	16.33		16.03	15.56		15.56	12.56		12.56	15.45	15.42		15.20	15.52		16.25	15.35		15.85	15.20
**M19-MEL**		10.74	15.10		16.05	15.03		16.60	12.88		12.88	17.02	15.52		15.20	15.38		15.30	15.20		15.27	15.13
**MALME-3M**		21.98	14.55		7.03	6.92		7.03	6.02		6.02	7.18	7.21		7.31	7.31		7.21	7.24		7.24	7.14
**MDA-MB-435**		31.40	9.29		9.68	8.07		9.68	8.89		8.89	9.75	9.09		9.08	9.08		9.08	9.12		9.12	9.20
**MDA-N**		17.86	18.15		18.45	18.45		18.49	13.24		13.24	18.49	18.53		18.53	18.53		18.53	18.53		18.53	18.28
**SK-MEL-28**		21.33	7.46		5.22	5.10		5.14	4.28		4.28	4.81	4.58		4.77	4.82		4.79	4.88		4.83	4.83
**SK-MEL-2**		24.21	12.85		13.90	12.05		14.52	17.02		17.02	14.59	14.62		14.82	14.82		14.89	14.89		14.69	14.79
**SK-MEL-5**		11.40	18.32		16.98	15.67		15.67	12.08		12.08	15.81	15.88		16.48	16.40		16.86	17.99		18.20	18.32
**UACC-257**		47.97	9.77		6.74	6.89		7.29	8.30		8.30	7.52	7.31		7.26	7.29		7.36	7.40		7.05	7.05
**UACC-62**		22.75	12.91		7.57	7.41		7.46	11.04		11.04	11.04	7.45		7.52	6.55		6.50	6.52		6.59	6.65
**A549-ATCC**		19.14	26.85		35.24	31.77		32.58	33.88		33.88	33.34	33.65		34.12	37.84		37.83	37.50		37.76	38.55
**EKVX**		30.76	37.84		18.28	23.77		23.44	16.57		16.57	23.01	23.01		32.01	22.96		23.12	23.23		23.23	23.23
**HOP-18**		22.23	3.91		4.02	4.09		4.15	4.50		4.50	5.15	4.11		4.33	4.33		4.40	4.42		4.37	4.37
**HOP-62**		15.74	17.94		12.36	14.00		13.71	12.33		12.33	13.88	13.12		13.24	13.64		13.61	13.58		13.99	14.19
**HOP_92**		6.78	6.37		3.74	4.41		4.95	3.19		3.19	4.77	5.24		5.23	4.99		5.18	5.55		6.16	6.21
**LXFL-529**		17.06	6.19		10.26	10.07		9.61	10.18		10.18	9.27	8.45		8.91	7.85		7.36	6.73		6.51	6.68
**NCI-H226**		33.50	25.94		17.22	17.70		17.95	15.07		15.07	17.78	17.82		17.90	17.99		18.11	18.15		18.07	17.54
**NCI-H23**		11.91	13.21		15.24	11.53		11.04	15.81		15.81	10.08	8.99		8.71	7.99		7.73	7.41		7.34	7.21
**NCI-H322M**		20.80	15.24		20.00	18.28		20.41	21.98		21.98	20.51	20.65		20.80	20.70		20.28	20.28		20.46	19.86
**NCI-H460**		16.60	4.00		10.91	9.79		10.47	5.59		5.59	10.47	10.40		10.54	10.54		10.45	10.54		10.37	10.45
**NCI-H522**		30.38	20.04		26.06	23.24		26.55	26.12		26.12	26.85	27.80		26.79	27.92		28.64	28.05		28.05	27.48
**IGROV1**		19.54	22.13		17.18	13.21		13.58	16.56		16.56	13.87	13.93		13.93	13.96		14.16	14.09		14.16	14.39
**NCI-ADR-RES**		24.49	15.92		3.10	3.89		4.33	7.73		7.73	4.24	4.25		4.20	4.10		4.04	4.04		4.00	3.99
**OVCAR-3**		15.24	13.37		10.35	10.66		10.00	9.70		9.70	9.95	9.97		10.00	9.73		9.68	9.51		9.42	9.31
**OVCAR-4**		17.50	19.05		15.45	14.96		14.29	11.45		11.45	14.96	14.65		14.55	14.26		14.35	15.03		15.31	15.45
**OVCAR-5**		23.39	36.90		21.63	17.86		21.43	19.36		19.36	21.13	20.80		21.18	21.48		21.43	22.23		22.54	22.85
**OVCAR-8**		17.26	15.85		6.55	7.62		7.59	16.29		16.29	8.36	9.16		9.35	8.85		8.69	8.34		8.00	8.28
**SK-OV-3**		22.96	30.34		20.28	22.33		24.15	28.71		28.71	23.39	24.77		26.12	26.30		26.73	26.67		26.00	25.82
**DU-145**		11.45	11.12		3.03	2.62		2.10	6.84		6.84	2.18	2.15		2.12	2.04		2.02	2.02		2.03	1.99
**PC-3**		15.49	14.39		16.14	12.79		17.91	16.52		16.51	18.24	17.91		17.02	16.18		16.07	15.85		16.11	16.22
**786-0**		18.20	19.23		24.72	25.55		25.18	23.33		23.33	24.43	24.27		24.15	23.33		23.66	22.59		21.68	21.13
**A498**		29.85	10.23		10.18	7.45		6.81	10.37		10.37	6.93	6.90		7.19	7.11		7.06	7.08		6.92	6.87
**ACHN**		20.00	23.55		15.42	15.74		15.92	12.25		12.25	16.11	16.11		16.11	16.11		15.96	15.88		15.99	15.74
**CAKI-1**		27.29	9.10		7.66	6.50		6.52	9.75		9.75	6.02	5.71		5.29	5.13		5.02	4.95		4.92	4.81
**RXF-393**		7.69	2.55		1.60	1.70		1.65	2.46		2.46	1.81	1.99		2.05	2.03		2.09	2.09		2.17	2.20
**RXF-631**		28.51	2.73		5.28	5.01		4.58	4.57		4.57	4.65	4.51		4.55	4.55		4.55	4.52		4.54	4.59
**SN12C**		21.53	11.59		4.77	4.83		4.84	7.57		7.57	4.81	5.08		5.37	5.44		5.47	5.52		5.37	5.37
**SN12K1**		3.89	0.03		0.03	0.03		0.03	0.03		0.03	0.03	0.03		0.03	0.03		0.03	0.03		0.03	0.03
**TK-10**		27.73	18.84		16.56	17.74		20.51	16.90		16.90	22.18	24.32		25.94	27.23		27.48	2.73		28.64	28.71
**UO-31**		13.37	20.28		13.96	13.49		14.93	21.68		21.68	14.59	14.49		14.26	13.06		13.58	12.88		12.79	13.06
**DMS-114**		12.53	19.86		11.99	1.88		2.25	11.17		11.17	1.93	1.73		1.75	1.72		1.68	1.67		1.67	1.67
**DMS-273**		28.64	13.35		4.36	4.32		4.07	7.53		7.53	4.61	5.00		5.22	5.89		6.68	8.36		9.35	8.93

**Legend**: **IC_50_**—half maximal inhibitory concentration. Breast cancers: BT-549, HS-578T, MCF-7, MDA-MB-231-AT, MDA-MB-468, T-47D. Central Nerve System cancers: SF-268, SF-295, SF-539, SNB-19, SNB-75, SNB-78, U251, XF-498. Colon cancers: COLO-205, DLD-1, HCC-2998, HCT-116, HCT-15, HT29, KM112, KM20L2, SW-620. Leukemia: CCR-CEM, HL-60TB, K-562, MOLT-4, P388-ADR, P388-ADR, P388, RPMI-8226, SR. Melanoma: LOX-IMVI, M14, M19-MEL, MALME-3M, MDA-MB-435, MDA-N, SK-MEL-28, SK-MEL-2, SK-MEL-5, UACC-257, UACC-62. Non Small Cell Lung cancers: A549-ATCC, EKVX, HOP-18, HOP-62, HOP_92, LXFL-529, NCI-H226, NCI-H23, NCI-H322M, NCI-H460, NCI-H522. Ovarian cancers: IGROV1, NCI-ADR-RES, OVCAR-3, OVCAR-4, OVCAR-5, OVCAR-8, SK-OV-3. Prostate cancers: DU-145, PC-3. Renal cancers: 786-0, A498, ACHN, CAKI-1, RXF-393, RXF-631, SN12C, SN12K1, TK-10, UO-31. Small Cell Lung cancers: DMS-114, DMS-273.

**Table 2:** The outcomes from CB-Dock2 web server, top 5 cavities from largest, C1, to smallest, C5, accompanied by their respective calculated volumes (Å^3^), along with the coordinates of their centres and sizes in angstroms (Å).

CurPocket ID	Cavity Volume (Å^3^)	Center (x, y, z)	Cavity Size (x, y, z)
**C1**	818	38, −3, 25	15, 13, 13
**C2**	161	35, −14, 34	8, 11, 4
**C3**	96	38, 13, −3	8, 8, 5
**C4**	95	41, −19, 20	7, 4, 6
**C5**	84	32, 18, 19	5, 8, 10

**Table 3:** The optimal docking outcomes for each cavity with OADs **2f** and **2e**, with 1MP8.

CurPocket ID	Dimer	Vina Score (kcal⋅mol^−1^)	Cavity Volume (Å^3^)	Center (x, y, z)	Cavity Size (x, y, z)	Docking Size (x, y, z)
**C1**	**2f**	**−11.6**	818	38, −3, 25	15, 13, 13	30, 30, 30
**C2**	**2e**	**−8.6**	161	35, −14, 34	8, 11, 4	30, 30, 30

**Table 4:** Comparison of IC_50_ values for OADs **2b**, **2d**, **2h**, and **2j** known from the literature [29,61].

≥30.01	20.01–30.00	15.01–20.00		10.01–15.00	5.01–10.00		1.01–5.00		0.10–0.99	0.01–0.09	
							
Cell Line	Compound Number IC_50_ [µM]										Lit.
	2b [29]/11a [61]		2d [29]/11b [61]			2h [29]/11c [61]		2j [29]/11d [61]			
**Hep-G2**	0.73 (0.06)		5.70 (0.31)			6.15 (0.57)		3.99 (0.02)			[61]
**A549**	<0.1		6.31 (0.55)			0.51 (0.05)		0.71 (0.07)			[61]
**BGC-823**	6.69 (0.59)		1.49 (0.09)			3.89 ± 0.03		48.34 (2.98)			[61]
**MCF-7**	4.74 (0.23)		<0.1			30.80 (4.29)		<0.1			[61]
**PC-3**	1.76 (0.15)		7.69 (0.81)			33.24 (2.44)		6.36 (0.56)			[61]
**SKBR-3**	6.67 (0.11)		1.12 (0.03)			6.02 (0.05)		9.99 (0.04)			[29]
**SKOV-3**	6.49 (0.01)		1.56 (0.01)			5.39 (0.02)		10.27 (0.05)			[29]
**PC-3**	6.43 (0.03)		1.64 (0.01)			5.34 (0.07)		9.81 (0.02)			[29]
**U-87**	6.59 (0.01)		1.20 (0.11)			5.87 (0.09)		10.68 (0.04)			[29]

**Legend**: **IC_50_**—half maximal inhibitory concentration; **SD**—the standard deviation; **Hep-G2**—*hepatocellular carcinoma*; **A549**—*lung carcinoma*; **BGC-823**—*gastric carcinoma*; **MCF-7**—*breast carcinoma*; **PC-3**—*prostatic carcinoma*; **SKBR-3**—*human breast adenocarcinoma*; **SKOV-3**—*human ovarian cystadenocarcinoma*; **U-87**—*human glioblastoma*.

## Data Availability

All data concerning this paper are available in the manuscript body or the Supplementary Data.

## Supplementary Materials

The following supporting information can be dwoloaded at: https://www.mdpi.com/article/10.3390/molecules29153623/s1, Figure S1. Largest Cavity C1 of FAK (PBD ID: 1MP8), with calculated volume 818 Å^3^. Figure S2. Cavity C2 of FAK (PBD ID: 1MP8), with calculated volume 161 Å^3^. Figure S3. Cavity C3 of FAK (PBD ID: 1MP8), with calculated volume 96 Å^3^. Figure S4. Cavity C4 of FAK (PBD ID: 1MP8), with calculated volume 95 Å^3^. Figure S5. Cavity C5 of FAK (PBD ID: 1MP8), with calculated volume 84 Å^3^. Table S1. Vina score in kcal⋅mol^−1^. Table S2. ADMETox parameters for OADs **2a**–**2n**.
