# Supplementary material for: Exploring the Potential of Oleanolic Acid Dimers–Cytostatic and Antioxidant Activities, Molecular Docking, and ADMETox Profile
Source: Molecules. 2024 Jul 31;29(15):3623. doi: 10.3390/molecules29153623 (PMC11313909; doi:10.3390/molecules29153623)
Supplement: Supplementary file 1 [file molecules-29-03623-s001.zip › molecules-3115932. Gunther et al. SM.01. Molecular Docking.pdf]

molecules

Article

Exploring the Potential of Oleanolic Acid Dimers–Cytostatic and Antioxidant Activities, Molecular Docking, and ADMETox Profile

Andrzej Günther [1,*], Przemysław Zalewski [2,3], Szymon Sip [2] and Barbara Bednarczyk-Cwynar [1,4]

1 Department of Organic Chemistry, Faculty of Pharmacy, Poznan University of Medical Sciences, Collegium Pharmaceuticum 2 (CP.2), Rokietnicka Str. 3, 60-806 Poznan, Poland; bcwynar@ump.edu.pl
2 Department of Pharmacognosy and Biomaterials, Faculty of Pharmacy, Poznan University of Medical Sciences, Collegium Pharmaceuticum 1 (CP.1), Rokietnicka Str. 3, 60-806 Poznan, Poland; pzalewski@ump.edu.pl (P.Z.); szymonsip@ump.edu.pl (S.S.)
3 Department of Pharmacology and Phytochemistry, Institute of Natural Fibres and Medicinal Plants, Wojska Polskiego 71b, 60-630 Poznan, Poland
4 Center of Innovative Pharmaceutical Technology (CITF), Rokietnicka Str. 3, 60-806 Poznan, Poland
* Correspondence: andrzej.gunther@me.pl

Supplementary Materials 01. Cavities of 1MP8

Figure S1. Largest Cavity C1 of FAK (PBD ID: 1MP8), with calculated volume 818 Å^3.

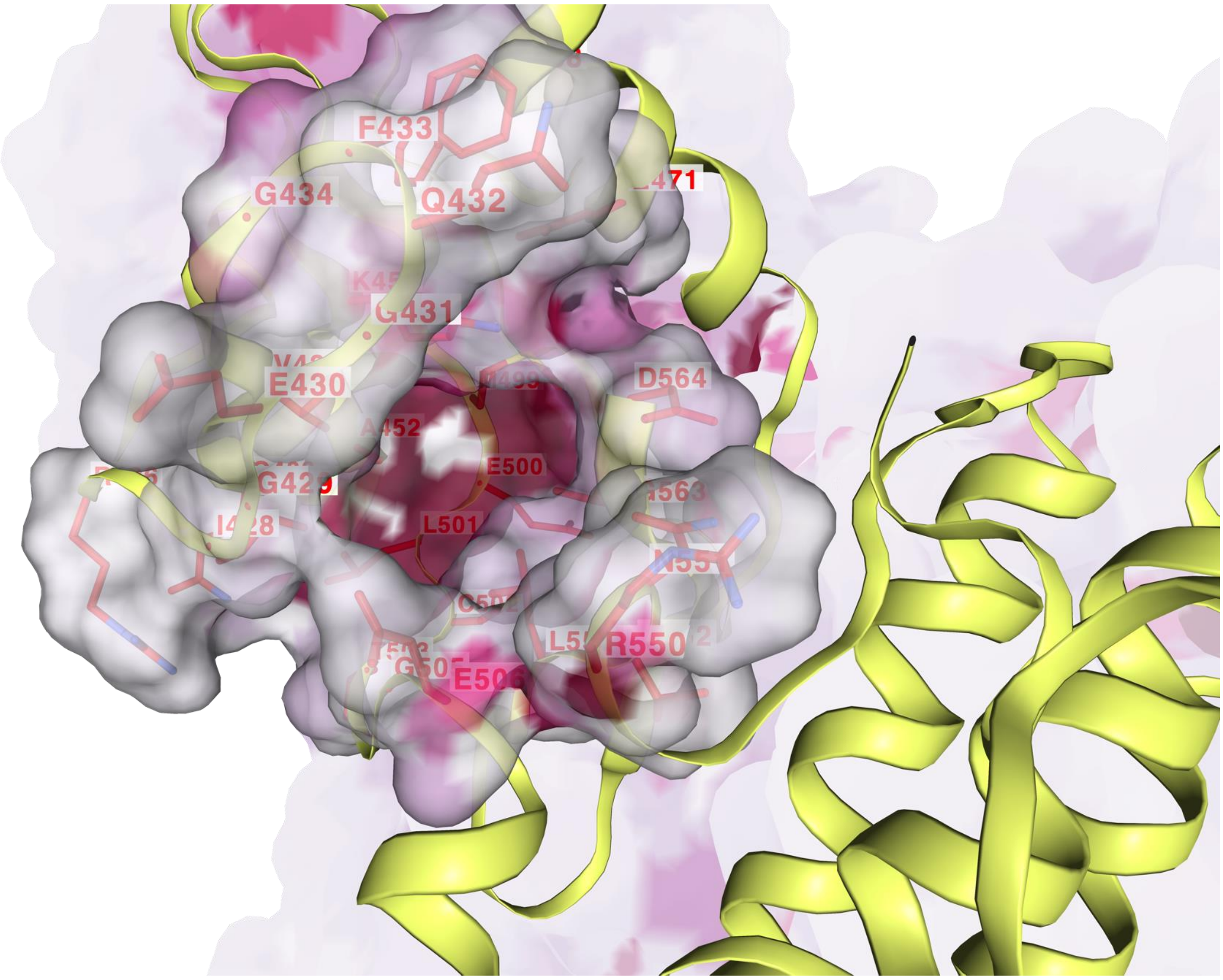

Figure S2. Cavity C2 of FAK (PBD ID: 1MP8), with calculated volume 161 Å^3.

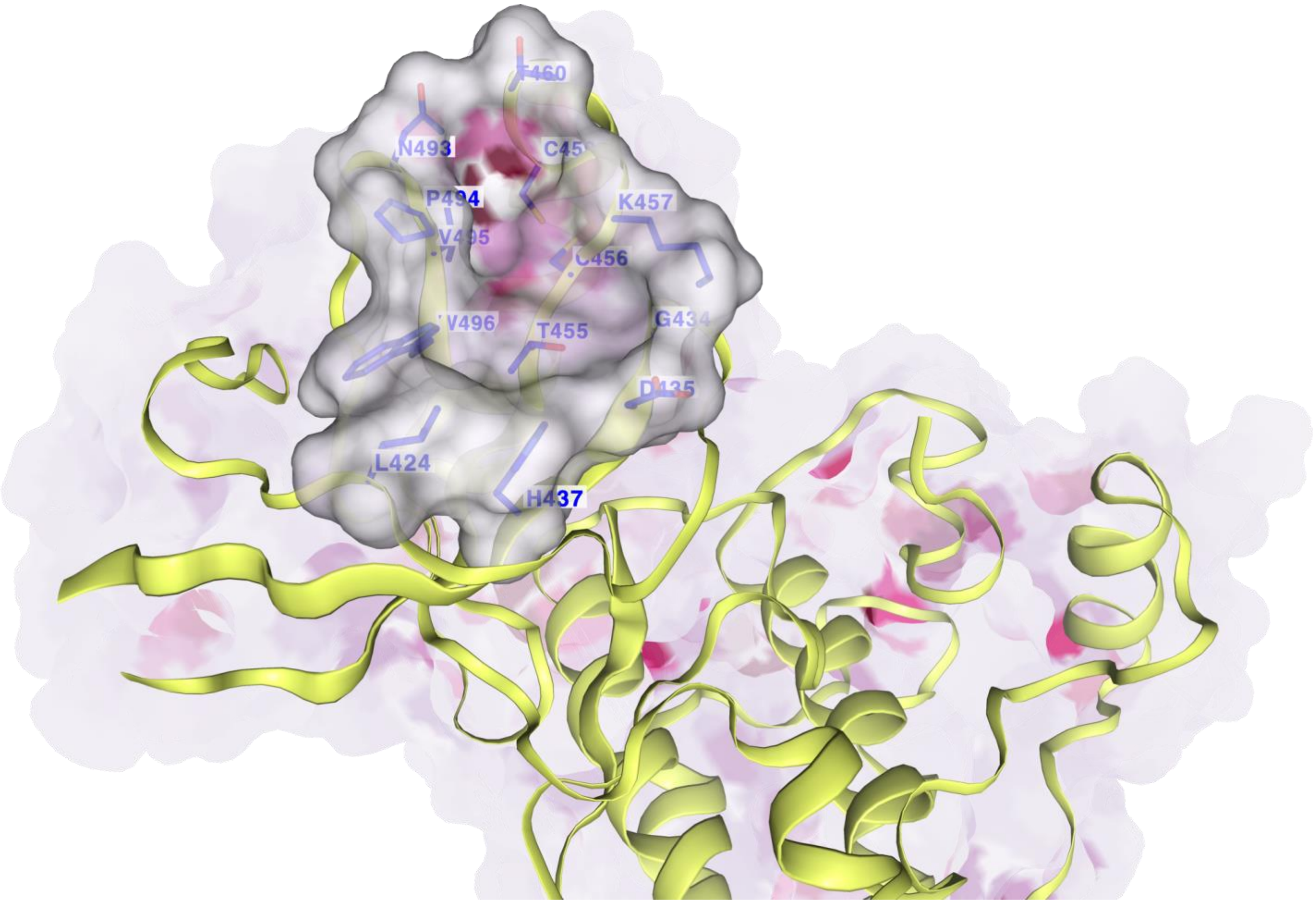

Figure S3. Cavity C3 of FAK (PBD ID: 1MP8), with calculated volume 96 Å^3.

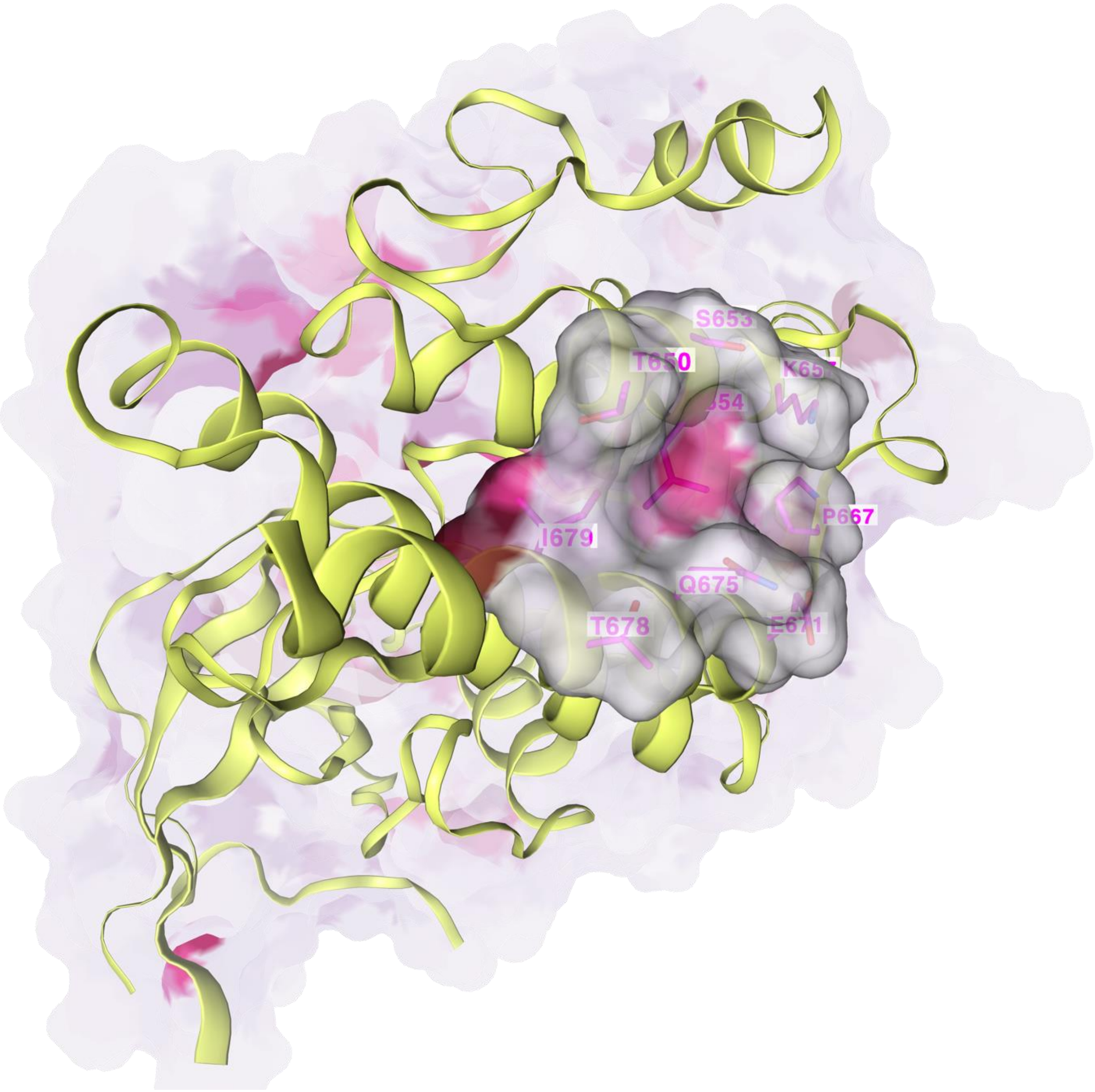

Figure S4. Cavity C4 of FAK (PBD ID: 1MP8), with calculated volume 95 Å^3.

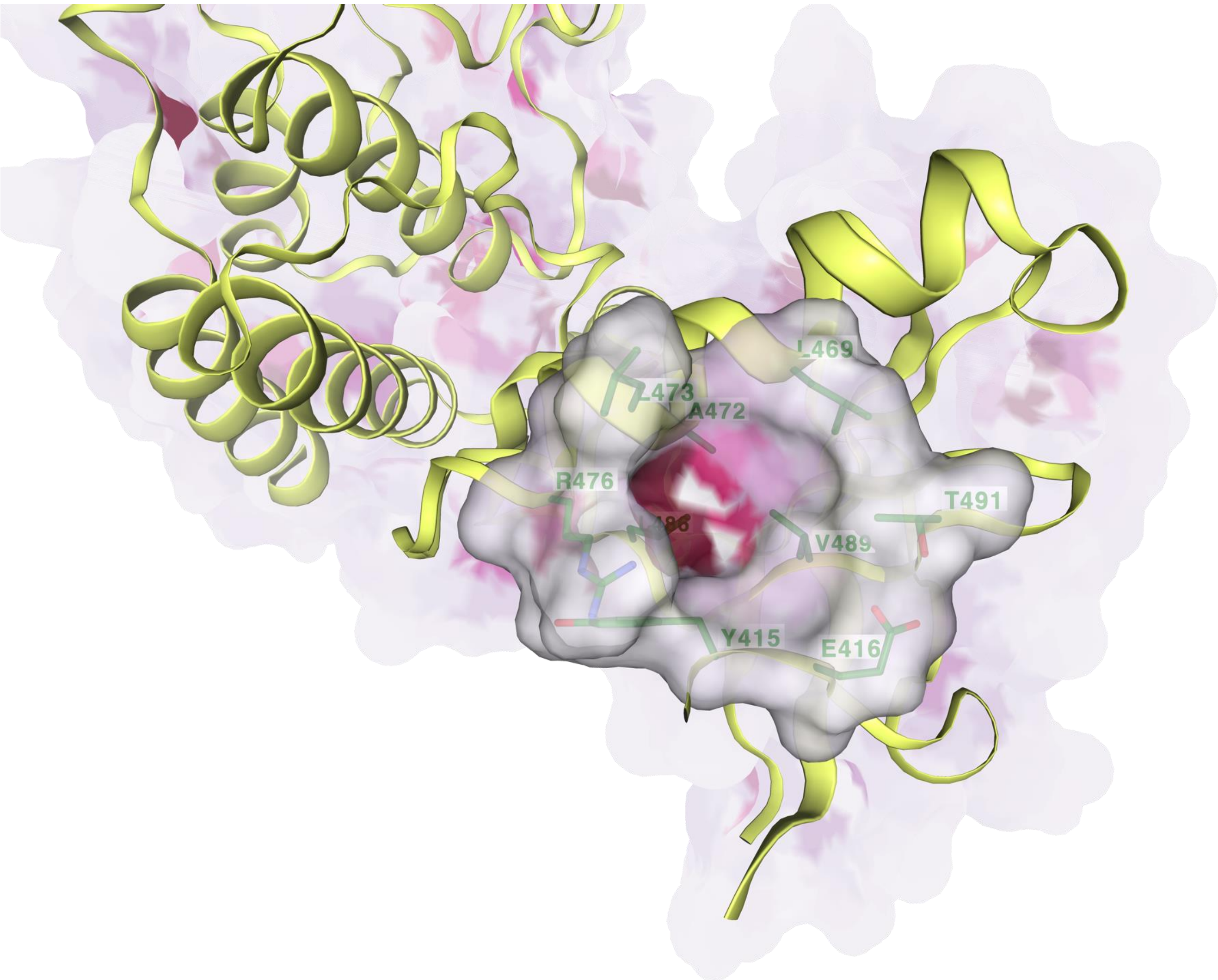

Figure S5. Cavity C5 of FAK (PBD ID: 1MP8), with calculated volume 84 Å^3.

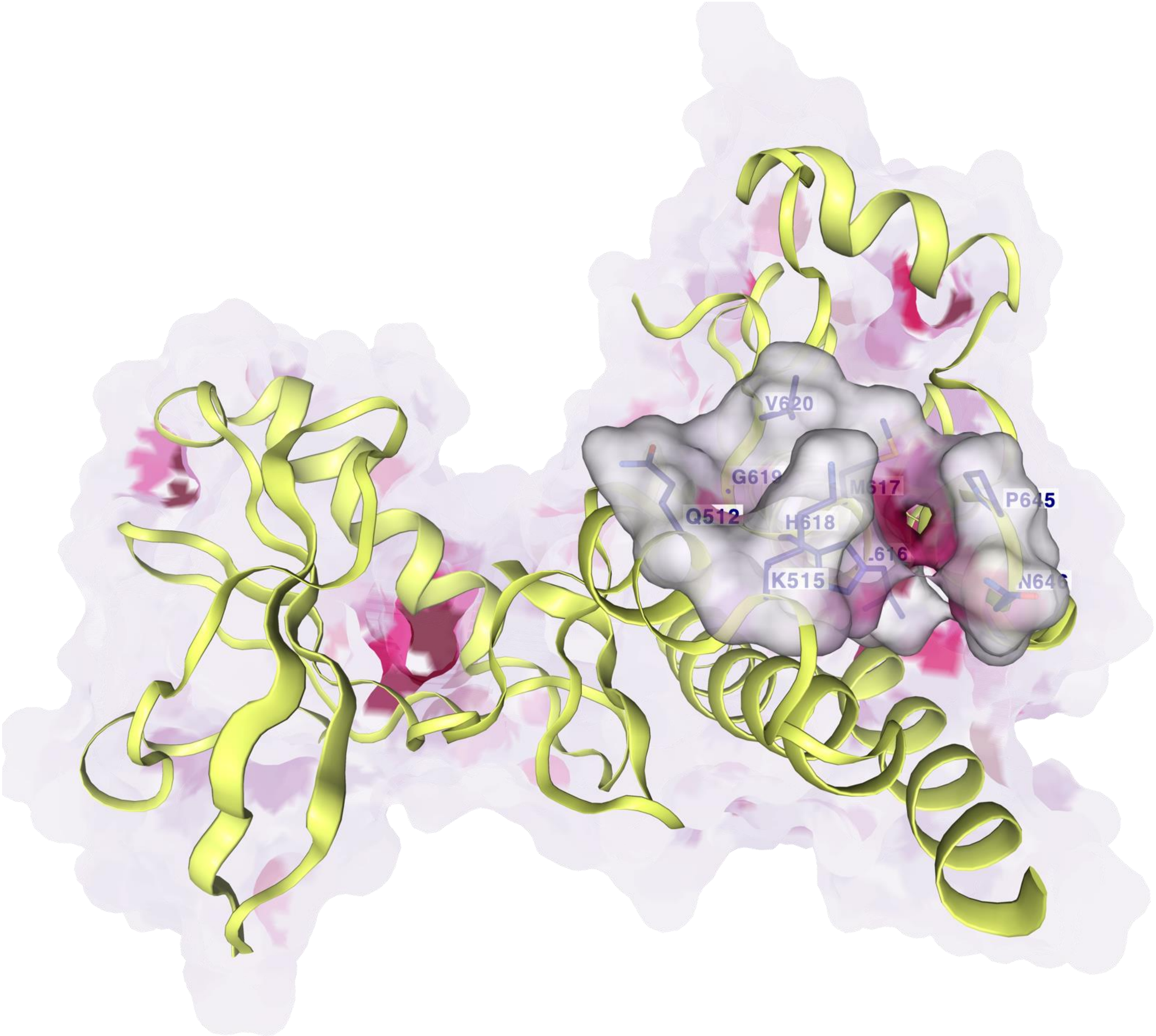

Table S1. Vina score in kcal · mol^{-1}

C1	C2	C3	C4	C5

Dimer No.	CurPocket ID	Vina score	Cavity volume (Å³)	Center (x, y, z)	Docking size (x, y, z)
2a	C1	-10.8	818	38, -3, 25	30, 30, 30
	C2	-8.6	96	38, 13, -3	30, 30, 30
	C3	-8.5	161	35, -14, 34	30, 30, 30
	C4	-7.9	95	41, -19, 20	30, 30, 30
	C5	-5.8	84	32, 18, 19	30, 30, 30
2b	C1	-9.8	818	38, -3, 25	29, 29, 29
	C2	-7.0	161	35, -14, 34	29, 29, 29
	C3	-7.1	96	38, 13, -3	29, 29, 29
	C4	-7.8	95	41, -19, 20	29, 29, 29
	C5	-6.6	84	32, 18, 19	29, 29, 29
2c	C1	-10.2	818	38, -3, 25	34, 34, 34
	C2	-7.9	161	35, -14, 34	34, 34, 34
	C3	-7.4	96	38, 13, -3	34, 34, 34
	C4	-8.5	95	41, -19, 20	34, 34, 34
	C5	-6.7	84	32, 18, 19	34, 34, 34
2d	C1	-9.6	818	38, -3, 25	30, 30, 30
	C2	-7.0	161	35, -14, 34	30, 30, 30
	C3	-8.2	96	38, 13, -3	30, 30, 30
	C4	-7.9	95	41, -19, 20	30, 30, 30
	C5	-7.4	84	32, 18, 19	30, 30, 30
2e	C1	-9.4	818	38, -3, 25	30, 30, 30
	C2	-8.6	161	35, -14, 34	30, 30, 30
	C3	-7.9	96	38, 13, -3	30, 30, 30
	C4	-8.7	95	41, -19, 20	30, 30, 30
	C5	-8.5	84	32, 18, 19	30, 30, 30
2f	C1	-11.6	818	38, -3, 25	30, 30, 30
	C2	-8.5	95	41, -19, 20	30, 30, 30
	C3	-7.5	161	35, -14, 34	30, 30, 30
	C4	-6.6	96	38, 13, -3	30, 30, 30
	C5	-5.7	84	32, 18, 19	30, 30, 30
2g	C1	-10.6	818	38, -3, 25	35, 35, 35
	C2	-7.4	161	35, -14, 34	35, 35, 35
	C3	-7.2	95	41, -19, 20	35, 35, 35
	C4	-6.5	96	38, 13, -3	35, 35, 35
	C5	-5.9	84	32, 18, 19	35, 35, 35
2h	C1	-9.6	818	38, -3, 25	36, 36, 36
	C2	-7.2	161	35, -14, 34	36, 36, 36
	C3	-7.5	96	38, 13, -3	36, 36, 36
	C4	-8.6	95	41, -19, 20	36, 36, 36
	C5	-7.9	84	32, 18, 19	36, 36, 36
2i	C1	-7.6	818	38, -3, 25	37, 37, 37
	C2	-8.0	161	35, -14, 34	37, 37, 37
	C3	-6.2	96	38, 13, -3	37, 37, 37

	C4	-7.9	95	41, -19, 20	37, 37, 37
	C5	-6.8	84	32, 18, 19	37, 37, 37
**2j**	C1	-10.4	818	38, -3, 25	36, 36, 36
	C2	-7.4	161	35, -14, 34	36, 36, 36
	C3	-7.0	96	38, 13, -3	36, 36, 36
	C4	-7.7	95	41, -19, 20	36, 36, 36
	C5	-6.9	84	32, 18, 19	36, 36, 36
**2k**	C1	-8.4	818	38, -3, 25	39, 39, 39
	C2	-6.1	161	35, -14, 34	39, 39, 39
	C3	-6.8	96	38, 13, -3	39, 39, 39
	C4	-7.0	95	41, -19, 20	39, 39, 39
	C5	-7.4	84	32, 18, 19	39, 39, 39
**2l**	C1	-9.4	818	38, -3, 25	39, 39, 39
	C2	-6.6	161	35, -14, 34	39, 39, 39
	C3	-5.7	96	38, 13, -3	39, 39, 39
	C4	-7.8	95	41, -19, 20	39, 39, 39
	C5	-6.1	84	32, 18, 19	39, 39, 39
**2m**	C1	-6.4	818	38, -3, 25	43, 43, 43
	C2	-8.2	161	35, -14, 34	43, 43, 43
	C3	-5.1	96	38, 13, -3	43, 43, 43
	C4	-7.3	95	41, -19, 20	43, 43, 43
	C5	-5.4	84	32, 18, 19	43, 43, 43
**2n**	C1	-8.6	818	38, -3, 25	44, 44, 44
	C2	-6.8	161	35, -14, 34	44, 44, 44
	C3	-5.8	96	38, 13, -3	44, 44, 44
	C4	-8.2	95	41, -19, 20	44, 44, 44
	C5	-6.3	84	32, 18, 19	44, 44, 44
