# Supplementary material for: Exploring the Potential of Oleanolic Acid Dimers–Cytostatic and Antioxidant Activities, Molecular Docking, and ADMETox Profile
Source: Molecules. 2024 Jul 31;29(15):3623. doi: 10.3390/molecules29153623 (PMC11313909; doi:10.3390/molecules29153623)
Supplement: Supplementary file 1 [file molecules-29-03623-s001.zip › molecules-3115932. Gunther et al. SM.02. ADMETox data.pdf]

molecules

MDPI

Article

Exploring the Potential of Oleanolic Acid Dimers–Cytostatic and Antioxidant Activities, Molecular Docking, and ADMETox Profile

Andrzej Günther [1,*], Przemysław Zalewski [2,3], Szymon Sip [2] and Barbara Bednarczyk-Cwynar [1,4]

[1] Department of Organic Chemistry, Faculty of Pharmacy, Poznan University of Medical Sciences, Collegium Pharmaceuticum 2 (CP.2), Rokietnicka Str. 3, 60-806 Poznan, Poland; bcwynar@ump.edu.pl
[2] Department of Pharmacognosy and Biomaterials, Faculty of Pharmacy, Poznan University of Medical Sciences, Collegium Pharmaceuticum 1 (CP.1), Rokietnicka Str. 3, 60-806 Poznan, Poland; pzalewski@ump.edu.pl (P.Z.); szymonsip@ump.edu.pl (S.S.)
[3] Department of Pharmacology and Phytochemistry, Institute of Natural Fibres and Medicinal Plants, Wojska Polskiego 71b, 60-630 Poznan, Poland
[4] Center of Innovative Pharmaceutical Technology (CITF), Rokietnicka Str. 3, 60-806 Poznan, Poland
* Correspondence: andrzej.gunther@me.pl

Supplementary Materials

Table S2. ADMETox parameters for OADs 2a – 2n

High value (optimal)	Moderate value	Low value	Neutral value

**ADMETox parameters** (optimal values)	**Compound number**														
	**1 (OA)**	**2a**	**2b**	**2c**	**2d**	**2e**	**2f**	**2g**	**2h**	**2i**	**2j**	**2k**	**2l**	**2m**	**2n**
Molecular Weight (100 ~ 600)	456.36	928.75	938.74	952.75	966.77	964.75	964.75	980.78	994.80	1008.81	1022.83	1036.85	1050.86	1064.88	1078.89
Volume	505.75	1025.52	1037.54	1054.83	1072.13	1069.50	1069.49	1089.43	1106.72	1124.02	1141.31	1158.61	1175.91	1193.20	1210.50
Density	0.902	0.906	0.905	0.903	0.902	0.902	0.902	0.900	0.899	0.898	0.896	0.895	0.894	0.892	0.891
NHA (0 ~ 12)	3	6	6	6	6	6	6	6	6	6	6	6	6	6	6
nHD (0 ~ 7)	2	4	2	2	2	2	2	2	2	2	2	2	2	2	2
nRot (0 ~ 11)	1	6	7	8	9	8	8	10	11	12	13	14	15	16	17

nRing (0 ~ 6)	5	10	10	10	10	10	10	10	10	10	10	10	10	10	10
maxRing (0 ~ 18)	22	22	22	22	22	22	22	22	22	22	22	22	22	22	22
nHet (1 ~ 15)	3	6	6	6	6	6	6	6	6	6	6	6	6	6	6
fChar (-4 ~ +4)	0	0	0	0	0	0	0	0	0	0	0	0	0	0	0
nRig (0 ~ 30)	27	52	54	54	54	55	55	54	54	54	54	54	54	54	54
Flexibility (≤ 2)	0.037	0.115	0.13	0.148	0.167	0.145	0.145	0.185	0.204	0.222	0.241	0.259	0.278	0.296	0.315
Stereo Centers (≤ 2)	8	18	16	16	16	16	16	16	16	16	16	16	16	16	16
TPSA (0 ~ 140)	57.53	99.38	93.06	93.06	93.06	93.06	93.06	93.06	93.06	93.06	93.06	93.06	93.06	93.06	93.06
LogS (-4 ~ 0.5)	-5.036	-5.622	-7.378	-7.445	-7.508	-5.39	-6.896	-7.578	-7.648	-7.723	-7.804	-7.884	-7.96	-8.035	-8.104
LogP (0 ~ 3)	6.113	10.726	11.299	11.506	11.925	11.195	11.629	12.327	12.729	13.126	13.520	13.914	14.309	14.703	15.097
LogD (1 ~ 3)	4.843	6.297	6.910	6.932	7.004	6.966	7.007	7.103	7.199	7.290	7.381	7.472	7.563	7.654	7.745
QED (> 0.67)	0.409	0.156	0.162	0.157	0.150	0.203	0.203	0.143	0.136	0.129	0.122	0.115	0.108	0.102	0.096
Sascore (< 6)	4.589	6.457	6.06	6.098	6.103	6.214	6.214	6.124	6.135	6.146	6.159	6.171	6.185	6.199	6.213
Fsp3 (≥ 0.420)	0.900	0.934	0.903	0.905	0.906	0.875	0.875	0.908	0.909	0.910	0.912	0.913	0.914	0.915	0.917
MCE-18 (≥ 45)	105.368	208.881	208.881	208.0	207.148	208.0	208.0	206.323	205.52	204.75	204.0	203.27	202.57	201.88	201.217
Npscore (-5 ~ 5)	3.272	1.453	1.558	1.557	1.534	1.590	1.590	1.527	1.506	1.485	1.465	1.446	1.427	1.408	1.390
Lipinski Rule	A	R	R	R	R	R	R	R	R	R	R	R	R	R	R
Pfizer Rule	R	A	A	A	A	A	A	A	A	A	A	A	A	A	A
GSK Rule	R	R	R	R	R	R	R	R	R	R	R	R	R	R	R
Golden Triangle	A	R	R	R	R	R	R	R	R	R	R	R	R	R	R
PAINS (alerts)	0	0	0	0	0	0	0	0	0	0	0	0	0	0	0
ALARM NMR (alerts)	0	0	0	0	0	0	0	0	0	0	0	0	0	0	0
BMS (alerts)	0	2	0	0	0	0	0	0	0	0	0	0	1	1	1
Chelator Rule (alerts)	0	0	0	0	0	0	0	0	0	0	0	0	0	0	0
Caco-2 Permeability (> -5.15)	-5.198	-5.279	-5.222	-5.217	-5.225	-5.176	-5.236	-5.236	-5.249	-5.261	-5.269	-5.279	-5.289	-5.298	-5.308
MDCK Permeability (< 2×10^{-6} cm/s)	2×10^{-5}	6×10^{-6}	6×10^{-6}	6×10^{-6}	5×10^{-6}	7×10^{-6}	6×10^{-6}	5×10^{-6}	5×10^{-6}	5×10^{-6}	4×10^{-6}	4×10^{-6}	4×10^{-6}	4×10^{-6}	4×10^{-6}
Pgp Inhibitor	0.000	0.000	0.000	0.000	0.000	0.000	0.000	0.000	0.000	0.000	0.000	0.000	0.000	0.000	0.000

Pgp Substrate	0.000	0.000	0.000	0.000	0.000	0.000	0.000	0.000	0.000	0.000	0.000	0.000	0.000	0.000	0.000
HIA (< 30%)	0.012	0.581	0.769	0.804	0.806	0.405	0.756	0.782	0.743	0.690	0.643	0.592	0.533	0.472	0.395
$F_{20\%}$ (< 20%)	0.074	0.992	0.993	0.996	0.997	0.991	0.981	0.999	0.999	0.999	1.000	1.000	1.000	1.000	1.000
$F_{30\%}$ (< 30%)	0.756	0.968	0.990	0.993	0.995	0.954	0.990	0.996	0.997	0.998	0.998	0.998	0.999	0.999	0.999
PPB (< 90%)	98.13%	97.25%	94.99%	94.31%	93.77%	98.30%	98.16%	93.90%	93.91%	94.05%	94.32%	94.65%	95.01%	95.48%	96.03%
VD (0.04 – 20 L/kg)	0.718	1.194	1.100	1.083	1.094	1.188	1.071	1.100	1.108	1.125	1.146	1.166	1.187	1.211	1.255
BBB Penetration	0.674	0.593	0.367	0.335	0.350	0.713	0.419	0.358	0.366	0.373	0.379	0.39	0.401	0.409	0.415
Fu (> 20%)	3.524%	2.087%	2.070%	1.906%	1.718%	1.666%	2.011%	1.555%	1.418%	1.291%	1.179%	1.074%	0.984%	0.914%	0.850%
CYP1A2 Inhibitor	0.012	0.000	0.002	0.002	0.001	0.001	0.001	0.001	0.001	0.001	0.001	0.001	0.001	0.001	0.001
CYP1A2 Substrate	0.323	0.217	0.209	0.206	0.205	0.206	0.189	0.206	0.208	0.21	0.212	0.213	0.213	0.213	0.212
CYP2C19 Inhibitor	0.028	0.013	0.027	0.028	0.027	0.024	0.024	0.027	0.027	0.027	0.027	0.026	0.026	0.026	0.025
CYP2C19 Substrate	0.916	0.959	0.959	0.958	0.961	0.953	0.941	0.962	0.961	0.959	0.958	0.956	0.954	0.951	0.947
CYP2C9 Inhibitor	0.157	0.033	0.040	0.038	0.034	0.035	0.035	0.031	0.029	0.026	0.024	0.021	0.019	0.017	0.015
CYP2C9 Substrate	0.813	0.033	0.091	0.086	0.105	0.106	0.092	0.124	0.146	0.171	0.202	0.23	0.266	0.302	0.339
CYP2D6 Inhibitor	0.012	0.026	0.009	0.013	0.011	0.023	0.026	0.010	0.008	0.007	0.006	0.005	0.004	0.004	0.003
CYP2D6 Substrate	0.528	0.101	0.113	0.104	0.104	0.443	0.290	0.092	0.083	0.075	0.067	0.060	0.054	0.050	0.046
CYP3A4 Inhibitor	0.172	0.191	0.374	0.352	0.293	0.307	0.281	0.271	0.253	0.238	0.223	0.209	0.196	0.182	0.169
CYP3A4 Substrate	0.208	0.672	0.877	0.872	0.861	0.905	0.871	0.847	0.832	0.816	0.797	0.776	0.753	0.729	0.703
CL (>15 mL/min/kg)	3.094	7.004	10.943	10.569	10.099	9.568	7.953	9.745	9.452	9.148	8.848	8.55	8.262	7.973	7.737
T1/2 (< 3h)	0.023	0.000	0.000	0.000	0.000	0.000	0.000	0.000	0.000	0.000	0.000	0.000	0.000	0.000	0.000
hERG Blockers	0.004	0.063	0.249	0.262	0.313	0.190	0.292	0.329	0.347	0.367	0.387	0.408	0.425	0.432	0.438
H-HT	0.296	0.202	0.199	0.201	0.193	0.207	0.200	0.191	0.189	0.188	0.187	0.186	0.185	0.184	0.182
DILI	0.010	0.001	0.003	0.003	0.003	0.003	0.003	0.003	0.003	0.003	0.003	0.003	0.003	0.003	0.003
AMES Toxicity	0.008	0.003	0.010	0.010	0.006	0.017	0.003	0.006	0.005	0.005	0.005	0.005	0.005	0.005	0.005
Rat Oral Acute Toxicity	0.228	0.130	0.198	0.200	0.172	0.208	0.270	0.163	0.154	0.148	0.142	0.135	0.129	0.123	0.117
FDAMDD	0.909	0.962	0.954	0.958	0.957	0.965	0.98	0.957	0.957	0.957	0.957	0.957	0.957	0.957	0.957
Skin Sensitization	0.028	0.018	0.036	0.04	0.043	0.049	0.057	0.048	0.055	0.066	0.079	0.093	0.116	0.145	0.18

Carcinogencity	0.063	0.02	0.03	0.028	0.025	0.038	0.032	0.023	0.021	0.02	0.018	0.017	0.015	0.014	0.013
Eye Corrosion	0.012	0.003	0.003	0.003	0.003	0.003	0.003	0.003	0.003	0.003	0.003	0.003	0.003	0.003	0.003
Eye Irritation	0.084	0.011	0.034	0.028	0.025	0.024	0.033	0.026	0.027	0.028	0.029	0.030	0.031	0.032	0.034
Respiratory Toxicity	0.968	0.981	0.874	0.87	0.862	0.746	0.886	0.851	0.839	0.827	0.814	0.796	0.777	0.756	0.735
Bioconcentration Factors	1.944	1.599	1.000	0.893	0.836	0.958	1.152	0.766	0.695	0.622	0.549	0.476	0.403	0.333	0.263
IGC$_{50}$	5.021	6.303	6.306	6.348	6.449	6.403	6.525	6.549	6.648	6.747	6.846	6.945	7.044	7.142	7.241
LC$_{50}$FM	5.937	7.516	7.247	7.28	7.292	7.265	7.759	7.315	7.338	7.36	7.382	7.402	7.388	7.364	7.321
LC$_{50}$DM	6.337	7.516	7.422	7.449	7.474	7.491	7.589	7.469	7.466	7.465	7.466	7.468	7.471	7.473	7.476
NR-AR	0.369	0.011	0.002	0.003	0.002	0.002	0.002	0.002	0.002	0.002	0.002	0.001	0.001	0.001	0.001
NR-AR-LBD	0.273	0.984	0.950	0.945	0.946	0.958	0.960	0.942	0.939	0.935	0.932	0.928	0.925	0.921	0.917
NR-AhR	0.001	0.000	0.000	0.000	0.000	0.000	0.000	0.000	0.000	0.000	0.000	0.000	0.000	0.000	0.000
NR-Aromatase	0.759	0.313	0.343	0.329	0.277	0.295	0.320	0.253	0.231	0.210	0.191	0.173	0.156	0.142	0.130
NR-ER	0.412	0.916	0.882	0.825	0.806	0.735	0.727	0.777	0.748	0.727	0.708	0.688	0.673	0.662	0.653
NR-ER-LBD	0.593	0.984	0.986	0.983	0.984	0.978	0.974	0.983	0.982	0.980	0.979	0.977	0.975	0.973	0.970
NR-PPAR gamma	0.965	0.919	0.945	0.940	0.933	0.951	0.948	0.928	0.922	0.915	0.905	0.891	0.876	0.858	0.838
SR-ARE	0.556	0.284	0.390	0.351	0.321	0.397	0.582	0.306	0.290	0.275	0.261	0.247	0.234	0.222	0.211
SR-ATAD5	0.052	0.321	0.665	0.542	0.515	0.658	0.669	0.456	0.403	0.353	0.308	0.266	0.229	0.194	0.163
SR-HSE	0.747	0.173	0.843	0.822	0.808	0.889	0.899	0.799	0.789	0.780	0.770	0.759	0.749	0.736	0.720
SR-MMP	0.971	0.988	0.945	0.935	0.909	0.946	0.968	0.891	0.869	0.841	0.809	0.772	0.728	0.678	0.624
SR-p53	0.271	0.124	0.402	0.293	0.229	0.380	0.426	0.182	0.142	0.115	0.092	0.070	0.050	0.036	0.025
Acute Toxicity Rule (alerts)	0	0	0	0	0	0	0	0	0	0	0	0	0	0	0
Genotoxic Carcinogenicity Rule (alerts)	0	0	0	0	0	1	1	0	0	0	0	0	0	0	0
Non Genotoxic Carcinogenicity Rule (alerts)	0	0	0	0	0	0	0	0	0	0	0	0	0	0	0
Skin SensitizationRule (alerts)	0	1	0	0	0	2	2	0	0	0	0	0	0	0	0
Aquatic Toxicity Rule (alerts)	1	2	1	1	1	1	1	1	1	1	1	1	1	1	1
Non Biodegradable Rule (alerts)	0	1	0	0	0	0	0	0	0	0	0	0	0	0	0

Legend: OA – oleanolic acid (reference compound);

For values given in a range 0.000 – 1.000 (unless stated otherwise): **0.000 – 0.300**: low probability, **0.301 – 0.699**: moderate probability, **0.700 – 1.000**: high probability

Additional Information: detailed information on individual ADMETox parameters are given in our earlier publication [xxx].
Physicochemical Properties: Molecular Weight, Volume, Density, nHA, nHD, nRot, nRing, MaxRing, nHet, fChar, nRig, Flexibility, tPSA, logS, logP, logD.
Medicinal Chemistry: QED, SAscore, Fsp3, MCE-18, Npscore, Lipinski Rule, Pfizer Rule, GSK Rule, Golden Triangle, PAINS, ALARM NMR, BMS, Chelator Rule.
Absorption: Caco-2 Permeability, MDCK Permeability, Pgp Inhibitor, Pgp Substrate, HIA, F20% , F30%.
Distribution: PPB, VD, BBB Penetration, Fu.
Metabolism: CYP1A2 Inhibitor, CYP1A2 Substrate, CYP2C19 Inhibitor, CYP2C19 Substrate, CYP2C9 Inhibitor, CYP2C9 Substrate, CYP2D6 Inhibitor, CYP2D6 Substrate, CYP3A4 Inhibitor, CYP3A4 Substrate.
Excretion: CL, T1/2.
Toxicity: hERG Blockers, H-HT, DILI, AMES ToxicityRat Oral Acute Toxicity, FDAMDD, Skin Sensitization, Carcinogenicity, Eye Corrosion, Eye Irritation, Respiratory Toxicity.
Environmental toxicity: Bioconcentration Factors, IGC50, LC50FM, LC50DM.
Tox21 pathway: NR-AR, NR-AR-LBD, NR-AhR, NR-ER, NR-ER-LBD, NR-PPARgamma, SR-ARE, SR-ATAD5, SR-HSE, SR-MMP.
Toxicophore Rules: Acute Toxicity Rule, Genotoxic Carcinogenicity Rule, NonGenotoxic Carcinogenicity Rule, Skin Sensitization Rule, Aquatic Toxicity Rule, NonBiodegradable Rule, SureChEMBL Rule.
